# Medicinal Plants Used in the Treatment of Human Immunodeficiency Virus

**DOI:** 10.3390/ijms19051459

**Published:** 2018-05-14

**Authors:** Bahare Salehi, Nanjangud V. Anil Kumar, Bilge Şener, Mehdi Sharifi-Rad, Mehtap Kılıç, Gail B. Mahady, Sanja Vlaisavljevic, Marcello Iriti, Farzad Kobarfard, William N. Setzer, Seyed Abdulmajid Ayatollahi, Athar Ata, Javad Sharifi-Rad

**Affiliations:** 1 Medical Ethics and Law Research Center, Shahid Beheshti University of Medical Sciences, 88777539 Tehran, Iran; bahar.salehi007@gmail.com; 2 Student Research Committee, Shahid Beheshti University of Medical Sciences, 22439789 Tehran, Iran; 3 Department of Chemistry, Manipal Institute of Technology, Manipal University, Manipal 576104, India; nv.anil@manipal.edu; 4 Department of Pharmacognosy, Gazi University, Faculty of Pharmacy, 06330 Ankara, Turkey; bilgesener11@gmail.com (B.Ş.); klcmehtap89@gmail.com (M.K.); 5 Department of Medical Parasitology, Zabol University of Medical Sciences, 61663-335 Zabol, Iran; 6 PAHO/WHO Collaborating Centre for Traditional Medicine, College of Pharmacy, University of Illinois, 833 S. Wood St., Chicago, IL 60612, USA; g.b.mahady@gmail.com; 7 Department of Chemistry, Biochemistry and Environmental Protection, Faculty of Sciences, University of Novi Sad, Trg Dositeja Obradovica 3, 21000 Novi Sad, Serbia; sanja.vlaisavljevic@dh.uns.ac.rs; 8 Department of Agricultural and Environmental Sciences, Milan State University, 20133 Milan, Italy; 9 Phytochemistry Research Center, Shahid Beheshti University of Medical Sciences, 11369 Tehran, Iran; farzadkf@yahoo.com (F.K.); majid_ayatollahi@yahoo.com (S.A.A.); 10 Department of Medicinal Chemistry, School of Pharmacy, Shahid Beheshti University of Medical Sciences, 11369 Tehran, Iran; 11 Department of Chemistry, University of Alabama in Huntsville, Huntsville, AL 35899, USA; 12 Department of Pharmacognosy, School of Pharmacy, Shahid Beheshti University of Medical Sciences, 11369 Tehran, Iran; 13 Department of Chemistry, Richardson College for the Environmental Science Complex, The University of Winnipeg, Winnipeg, MB R3B 2G3, Canada; a.ata@uwinnipeg.ca

**Keywords:** acquired immune deficiency syndrome, phytochemistry, pharmacognosy, antiviral, drug discovery

## Abstract

Since the beginning of the epidemic, human immunodeficiency virus (HIV) has infected around 70 million people worldwide, most of whom reside is sub-Saharan Africa. There have been very promising developments in the treatment of HIV with anti-retroviral drug cocktails. However, drug resistance to anti-HIV drugs is emerging, and many people infected with HIV have adverse reactions or do not have ready access to currently available HIV chemotherapies. Thus, there is a need to discover new anti-HIV agents to supplement our current arsenal of anti-HIV drugs and to provide therapeutic options for populations with limited resources or access to currently efficacious chemotherapies. Plant-derived natural products continue to serve as a reservoir for the discovery of new medicines, including anti-HIV agents. This review presents a survey of plants that have shown anti-HIV activity, both in vitro and in vivo.

## 1. Introduction

The World Health Organisation estimates that over 75 million people globally have been infected with the human immunodeficiency virus (HIV), of which approximately 37 million are still alive and living with the infection [1,2]. It is currently estimated that ~26 million of these patients reside in Africa; 3.3 million in the Americas; 3.5 million in Southeast Asia; 2.4 million in Europe; 360,000 in the eastern Mediterranean; and 1.5 million in the western Pacific [2]. Data from 2016 indicates that there were approximately two million new cases of HIV infections, and as many as one million deaths due to the disease [2]. Importantly, these annual numbers are much reduced, as the numbers of newly infected patients has declined by 35% since 2000, and the mortality rate has also declined by almost 50%. The decline in HIV infections is thought to be due to increased use of condoms, a reduction in the prevalence of sexually transmitted infection, and the increased use of effective therapies, such as the three-drug therapy anti-retroviral therapy (ART). The number of HIV patients now receiving antiretroviral therapy has increased from ~685,000 in 2000 to 20.9 million in 2017 [2].

While HIV is a significant cause of morbidity and mortality worldwide, the sub-Sahara region of Africa is burdened with the largest number of HIV cases [2]. Of the 37 million cases of HIV, the sub-Saharan Africa is home to ~70%, although it has only 21% of the world’s population. In fact, African men and women worldwide are more affected by this disease than any other race [2,3]. Only ten countries in southern and eastern Africa, including South Africa (25%), Nigeria (13%), Mozambique (6%), Uganda (6%), Tanzania (6%), Zambia (4%), Zimbabwe (6%), Kenya (6%), Malawi (4%) and Ethiopia (3%), account for approximately 80% of HIV patients [2,3]; In most countries, the prevalence of HIV is the highest in specific groups including men who have sex with men, intravenous drug users, people in prisons and other confined settings, sex workers and transgender individuals. However, unlike other countries, the primary HIV transmission mode in sub-Saharan Africa is through heterosexual sex, with a concomitant epidemic in children through vertical transmission [3]. As a consequence, African women are disproportionately affected and make up ~58% of the total number of people living with HIV, have the highest number of children living with HIV and the highest number of AIDS related deaths [2].

New data from coding complete genome analyses of US serum samples from 1978 to 1979 revealed that the US HIV-1 epidemic that occurred in the 1970s was extensively genetically diverse [4]. Bayesian phylogentic analyses of HIV-1 genomes suggest that the US epidemic emerged from a preexisting Caribbean epidemic with the place of the ancestral US virus being New York City [4]. The analysis of *gag*, *pol* and *env* RNA sequences placed the US sequences in a monophyletic clade nested within Caribbean subtype B sequences from Haiti, and other Caribbean countries, as well as Haitian immigrants in the US [4]. The data further suggested that the US clade emerged from the early growth phase of the Caribbean epidemic (1969–1973), which began after the introduction of the subtype B lineage from Africa about 1967 [4]. The Centers for Disease Control eventually made the connections between homosexual men with AIDS and Kaposi’s syndrome and sexual transmission of an infectious agent [5,6].

### 1.1. Pathophysiology

The HIV virus is a retrovirus that is able to integrate a DNA copy of the viral genome into the DNA of the host cells. The virus enters the cell through receptors that are expressed on the surface of T lymphocytes (activated T lymphocytes are preferred targets), monocytes, macrophages and dendritic cells [1,7]. To gain entry to the host cell, HIV-1 binds to the chemokine receptor 5 or the CXC chemokine receptor 4 through interactions with the envelope proteins. After fusion and uncoating, single stranded RNA is reverse transcribed into HIV DNA, and then integrated into the host DNA. HIV DNA is transcribed to viral mRNA and exported to the cytoplasm where it is translated to viral Gag, Gag-Pol, and Nef polyproteins, which are then cleaved later during virion assembly and maturation at the cell surface or after relase of the new viral particles. Current therapies inhibit many of the steps in this process, such as entry inhibitors, reverse transcriptase inhibitors, integrase strand transfer inhibitors and protease inhibitors [1,7].

### 1.2. Diagnosis

Detection of the HIV virus in the blood is usually measured as viral RNA load and infection is associated with an acute symptomatic period that includes fever, general malaise, lymphadenopathy, rash, myalgias, however serious consequences such as meningitis have also been reported [7,8]. During the period of acute infection, the plasma levels of HIV RNA are at their highest and the severity of symptoms is associated with the level of viral load. It has been suggested that viral characteristics and viral load determine both the replication and pathogenesis. Thus, the clinical outcomes and disease progression are dependent not only on the host, but also on the viral genotype [7]. HIV is difficult to completely eradicate as it establishes a quiescent or latent infection within the memory CD4^+^ T cells, which have a stem-cell-like capacity for self-renewal. Once the HIV DNA is integrated into the host chromatin, the virus can repeatedly initiate replication as long as that cell exists. While ART can prevent new cells from becoming infected, it cannot eliminate infection once the DNA has successfully integrated into the target cell. The lymph nodes harbor the virus because of limited antiretroviral drug penetration, and limited host clearance mechanisms, and serves as a source of virus recrudescence in individuals who stop or interrupt their therapy. It has been suggested that ART therapy may be needed for several decades before the viral reservoir might decay to negligible levels.

### 1.3. Current Treatments for HIV/AIDS

Although HIV was recognized early in the 1980s, there is still no cure or an effective vaccine for HIV infection, but there have been some significant advances in treatment, control, and prevention [9]. The introduction of anti-retroviral agents and highly active antiretroviral therapy (HAART) in 1996 significantly reduced the morbidity and mortality of HIV/AIDS. Antiretroviral therapy is currently recommended for all adults with HIV. Recommendations for initial regimens include two nucleoside reverse transcriptase inhibitors (NRTIs; abacavir with lamivudine or tenofovir disoproxil fumarate with emtricitabine) and an integrase strand transfer inhibitor, such as dolutegravir, elvitegravir, or raltegravir; a nonnucleoside reverse transcriptase inhibitor (efavirenz or rilpivirine) or a boosted protease inhibitor (darunavir or atazanavir) [10]. Alternative regimens are also available. Protease inhibitor monotherapy is generally not recommended, but NRTI-sparing approaches may be considered. Suspected treatment failure warrants rapid confirmation, performance of resistance testing while the patient is receiving the failing regimen, and evaluation of reasons for failure before consideration of switching therapy. Alterations in therapeutic regimens due to adverse effects, convenience, or to reduce costs should be carefully considered so as not to jeopardize antiretroviral potency. Research continues into HIV vaccines and antimicrobial agents, however other major advances in HIV prevention has been voluntary male medical circumcision [11,12], as well as antiretrovirals for the prevention of mother to child transmission [13,14,15,16].

The reduction in the morbidity and mortality of the disease has changed it from a fatal disease to a chronic, manageable condition [2,3,11,12]. Interestingly, the increased survival rate has resulted in an aging HIV/AIDS population, which has presented a whole new set of issues including a higher prevalence of chronic diseases in this population, such as cardiovascular and pulmonary diseases, malignancies and even a unique set of comorbidities, which are now designated as HIV-associated non-AIDS (HANA) conditions.

Antiretroviral agents remain the cornerstone of HIV treatment and prevention [17]. It is currently recommended that all HIV-infected patients with detectable virus, regardless of their CD4 cell count, should be treated with anti-retroviral therapy (ART) soon after diagnosis to prevent disease progression, improve clinical outcomes including reducing AIDS-associated events, non-AIDS-related events, and all-cause mortality, as well as to decrease transmission [17]. These recommendations are supported by large randomized controlled clinical trials it is recommended that all HIV-infected individuals with detectable plasma virus receive treatment with recommended initial regimens consisting of an integrase strand transfer inhibitors (InSTI) plus two nucleoside reverse transcriptase inhibitors (NRTIs). When used effectively, the anti-retroviral agents suppress HIV and prevent new HIV infections. It has been suggested that with these treatment regimens, that survival rates among HIV-infected adults can approach those of uninfected adults [17].

### 1.4. New Drug Therapies for HIV

A recent review of HIV therapies with new mechanisms of action in phase 2 clinical trials has reported on drugs with new mechanisms of action, including histone deacetylase (HDAC) inhibitors, gene therapies, broadly neutralizing anti-HIV antibodies, immune modulation, and drugs with new mechanisms to block HIV entry [18]. The new therapies are being developed for both as add-on therapy to existing combination antiretroviral therapy and as agents to be used during treatment interruption. The current drugs in development have had varying degrees of success in the early trials. Each of these new drugs may potentially fill a void in current antiretroviral therapy (ART) therapies, which will ultimately lead to improved outcomes in HIV-infected individuals.

### 1.5. Natural Products and Herbal Medicines for HIV

Although effective, ART is not without serious adverse events, which is especially evident in persons undergoing long-term treatment. In addition, the current therapies are limited by emergence of multidrug resistance [19], and new drugs and novel targets are needed to overcome the issues of HIV reservoirs in the body in order to have the complete eradication of HIV and AIDS. Latently infected cells remain a primary barrier to eradication of HIV-1. Over the last ten years the molecular mechanism by which HIV latency persists has led to the discovery of a number of drugs that are able to selectively reactivate latent proviruses without inducing polyclonal T cell activation [20]. Interestingly, histone deacetylase (HDAC) inhibitors, including vorinostat are able to induce HIV transcription from latently infected cells. Vorinostat has been shown to increase the susceptibility of CD4^+^ T cells to infection by HIV in a dose- and time-dependent manner, does not enhance viral fusion with cells, but increases reverse transcription, nuclear import, and integration, and enhances viral production in a spreading-infection assay. HDAC inhibitors, particularly vorinostat, are currently being investigated clinically as part of a “shock-and-kill” strategy to purge latent reservoirs of HIV [20].

Since new drugs will be needed for the management of HIV, the World Health Organization (WHO) has suggested the that ethnomedicines and other natural products should be systematically tested against HIV as they may yield effective and more affordable therapeutic agents (World Health Organization [21,22]. Interestingly, a significant amount of work in this area was performed in the 1990s, particularly investigations of natural products with activities against HIV-1 reverse transcriptase, HIV-1 and -2 proteases and integrases (extensively reviewed by Kurapati et al. [23]). The natural products calanolides (coumarins), ursolic and betulinic acids (triterpenes), baicalin (flavonoid), polycitone A (alkaloid), lithospermic acid (phenolic compound) have been proposed as promising candidates for anti-HIV agents [23]. However, most of these studies are in vitro, and too few investigations have been performed in vivo or in human studies. In terms of clinical data, a meta-analysis assessed 12 clinical trials involving 881 patients with AIDS to determine the efficacy of traditional Chinese medicines (TCM). The results showed that TCM interventions were associated with significantly reduced plasma viral load compared with placebo. This study further suggested that TCM interventions were significantly more effective than placebo for reducing plasma viral load and increasing CD4^+^ T lymphocyte count in patients with AIDS. However, when compared with conventional Western medicine, TCM interventions were significantly less effective in reducing viral load, but were associated with improved symptoms in a larger number of patients, with fewer adverse events [24]. Thus, there is significant potential for natural products and traditional medicines for the management of HIV infections and symptoms but in vivo and human studies are lacking.

## 2. Traditional Knowledge on Plants Used against HIV

Medicinal plants can be a promising alternative for various diseases and conditions [25,26,27,28,29,30,31,32,33,34,35,36,37,38,39,40,41,42,43,44,45,46]. The 717 species belonging to 151 families are reported in this article. The taxonomy of the plant species plays a significant role in the proper identification. The website, http://www.theplantlist.org and http://www.tropicos.org/Home.aspx were considered as the authentic sources of information in resolving the ambiguity of the names related to plants. A list of plant species with inhibition studies is summarized in Table 1. A majority of the inhibition studies are carried out on the crude extracts of the plant material by various solvents, while limited literature is available on the isolated natural products for different inhibition studies. Table 2 lists all the names which are reported in this article and their synonyms are reported in the literature.

The Food and Drug Administration (FDA or USFDA) classifies antiretroviral drugs for HIV infection into the following categories:

(1) Multi-class Combination Products,
(2) Nucleoside Reverse Transcriptase Inhibitors (NRTIs),
(3) Nonnucleoside Reverse Transcriptase Inhibitors (NNRTIs),
(4) Protease Inhibitors (PIs),
(5) Fusion Inhibitors,
(6) Entry Inhibitors—CCR5 co-receptor antagonist and
(7) HIV integrase strand transfer inhibitors.

For better understanding, 1st, 5th and 6th types are not explicitely mentioned in this article. 2nd and 3rd classes are categorized into HIV-reverse transcription (HIV-RT), 4th type as HIV-protease (HIV-PR) and 7th type as HIV-integrase (HIV-IN). Painter et al. [47] Konvalinka et al. [48] and Blanco et al. [49] have reviewed the roles of HIV-RT, HIV-PR and HIV-IN, respectively. Also, Matthée et al. [50] have discussed the natural inhibitors of HIV-RT.

Of these 717 species, HIV-RT, HIV-PR, and HIV-IN are reported for 206, 254 and 43 species, respectively. Apart from these three inhibitor studies, researchers have also evaluated 390 species for other enzyme inhibition studies which are grouped under anti-HIV activities.

## 3. Plant Extracts and Some Secondary Metabolites with Anti-HIV Activity

Most of the world’s cultures have centuries of tradition in the use of plant materials in order to control diseases. With recent advancement in pharmacognosy and technology along with the current trends of a more health-conscious general public, natural products are becoming a popular resource for researchers to discover novel and more effective antiviral drugs, considering the relatively reduced adverse effects and cost effectiveness of natural products in commercial scale [361]. Plants, as evolutionary responses to infections by fungi, nematodes, and other organisms, to avoid herbivory, and to comptete for light and space, produce numerous secondary metabolites such as phenolics, glycosides, alkaloids, coumarins, terpenoids, essential oils and peptides. These metabolites have been identified with different biological activities. Some of them play an important role in immune system enhancement, exhibiting antiviral potential [362], including viral infections associated with Human Immunodeficiency Virus type 1 (HIV-1) and 2 (HIV-2) as genetic variabilities. An increasing number of patients with HIV infection cannot use the currently approved anti-HIV drugs including the reverse transcriptase and protease inhibitors, due to the adverse reactions, particularly liver diseases, that have been reported for antiretroviral drugs. The best antiretroviral therapy (HAART) has also fallen short of completely suppressing HIV replication [363]. Therefore, the discovery and development of new anti-HIV agents or new mechanisms of activity from medicinal plants are required to reduce toxicity in drug application and to minimize side effects when compared with current synthetic drugs [364]. The potential utilization of plant extracts and their secondary metabolites to combat the development of anti-HIV agents is considered to be one of the most important approaches toward effective therapy for AIDS [365]. Bioassay-guided fractionation and isolation of secondary metabolites from medicinal plants according to their preliminary high throughput screenings provide systematic source to the novel compounds. The in vitro and in vivo evaluation affirmed the therapeutic potentials in these chemical compounds. Thus, traditional medicines can serve as sources of potential new drug candidates and initial research has focused on the isolation of bioactive lead compounds [366].

Many compounds with anti-HIV-1 effects have been screened and isolated from natural sources and discovered to inhibit HIV at nearly all stages of the viral life cycle. They include alkaloids, sulfated polysaccharides, polyphenolics, flavonoids, coumarins, phenolics, tannins, triterpenes, lectins, phloroglucinols, lactones, iridoids, depsidones, *O*-caffeoyl derivatives, lignans, ribosome inactivating proteins, saponins, xanthones, naphthodianthrones, photosensitisers, phosholipids, quinones and peptides [367]. Natural products provide a large reservoir for screening of anti-HIV agents with novel structures and anti-viral mechanisms because of their structural diversity. A variety of natural products have been found to inhibit unique enzymes and proteins crucial to the life cycle of HIV including efficient intervention with the reverse transcription process, virus entry, and integrase and protease inhibition [368]. However the mechanism of anti-HIV activities of many natural products is still unknown. Some of the plant extracts have significantly inhibited the enzyme activity of HIV-1 replication and protected cells infected with HIV-1. These extracts with anti-HIV activity are also active against other retroviruses such as Herpes Simplex Virus (HSV). Most studies have used in vitro test systems for anti-HIV-1 enzyme assays such as HIV-1 reverse transcriptase colorimetric assay, HIV-1 integrase assay, and HIV-1 protease fluorogenic assay, but a few in vivo studies have been carried out using compounds isolated from natural sources [369]. The anti-HIV activities of extracts from some medicinal plants have been reviewed.

### 3.1. Artemisia annua L. (Asteraceae)

The anti-HIV activity of the tea infusion prepared from the Chinese medicinal plant identified as *Artemisia annua* L. by using the validated cellular systems were examined. The tea infusion of *Artemisia annua* was found to be highly active with IC_50_ values as low as 2.0 μg/mL. In addition, artemisinin was found as inactive at 25 μg/mL and the related species *Artemisia afra* (not containing artemisinin) has also shown a similar level of activity [370].

### 3.2. Astragalus membranaceus Bunge (Fabaceae)

*Astragalus membranaceus* is well-known Chinese traditional medicine as an immunostimulant. Studies in immune-suppressed and immune-competent human patients have demonstrated restoration or augmentation of local graft versus host rejection using *Astragalus* extracts. These extracts have improved symptomology in HIV-infected patients. These results are suggested that the extracts of *Astragalus* to be safe, however mutagenecity has yet to be examined [115].

### 3.3. Calendula officinalis L. (Asteraceae)

In India, the flowers of *Calendula officinalis* are used in ointments for treating wounds, herpes, ulcers, frostbite, skin damage, scars and blood purification. The infusions prepared from the leaves have been used for treating varicose veins in traditional use. Dichloromethane-methanol (1:1) extract of *Calendula officinalis* flowers exhibited potent anti-HIV activity in in vitro (3-(4,5-dimethylthiazolyl-2)-2,5-diphenyltetrazolium bromide)(MTT)/tetrazolium-based assay. This activity was attributed to inhibition of HIV1-RT at a concentration of 1000 μg/mL as well as suppression of the HIV mediated fusion at 500 μg/mL [371]. The organic and aqueous extracts of dried flowers from *Calendula officinalis* were examined for their ability to inhibit the human immunodeficiency virus type 1 (HIV-l) replication. Both extracts were relatively nontoxic to human lymphocytic Molt-4 cells, but only the organic one exhibited potent anti-HIV activity in an in vitro MTT ketrazolium-based assay. In addition, in the presence of the organic extract (500 pg/mL), the uninfected Molt-4 cells were completely protected for up to 24 h from fusion and subsequent death, caused by cocultivation with persistently infected U-937/HIV-1 cells. It was also found that the organic extract from *Calendula officinalis* flowers caused a significant dose- and time-dependent reduction of HIV-l reverse transcription (RT) activity. An 85% RT inhibition was achieved after a 30 min treatment of partially purified enzyme in a cell-free system. These results suggested that organic extract of flowers from *Calendula oflicinalis* are possessed anti-HIV properties of therapeutic interest [163].

### 3.4. Calophyllum lanigerum Miq. var. austrocoriaceum (T.C. Whitmore) P.F. Stevens (Clusiaceae)

*Calophyllum lanigerum* var. *austrocoriaceum* has been found to inhibit the cytopathic effects of in vitro HIV infection. Bioassay-guided fractionation of the extract and the chemical along with biological characterization of active constituents as coumarine derivatives have been reported [372]. The latex of *Calophyllum teysmanni* L. has shown to be active against HIV-1 reverse transcriptase mediated by soulattrolide, a coumarin isolated from the latex of *Calophyllum teysmanni* [373].

### 3.5. Cassia abbreviata Oliv. Oliv., C. sieberiana D.C. (Fabaceae)

*Cassia abbreviata* growing in Botswana used by traditional healers to manage HIV/AIDS, was tested for their inhibitory effects on HIV replication against a clone of HIV-1c (MJ4) measuring cytopathic effect protection and levels of viral p24 antigen in infected PBMCs. *Cassia sieberiana* and *Cassia abbreviata* extracts have shown significant inhibition of HIV-1c (MJ4) replication. Anti-HIV activity of *Cassia sieberiana* root and bark extracts, and *Cassia abbreviata* root extracts were occurred in a concentration-dependent manner with an effective concentration (EC_50_) of 65.1 μg/mL, 85.3 μg/mL and 102.8 μg/mL, respectively [374].

### 3.6. Chelidonium majus L. (Papaveraceae)

The anti-retroviral activity of the freshly prepared crude extract of *Chelidonium majus* L. was examined and a low-sulfated poly-glycosaminoglycan moiety with molecular weight of ~3800 Da. was isolated from the extract [173]. The substance prevented infection of human CD4^+^ T-cell lines AA2 and H9 with HIV-1 at concentration of 25 μg/mL as well as the cell-to-cell virus spread in H9 cells continuously infected with HIV-1 were determined by the measurement of reverse transcriptase activity and p24 content in cell cultures. In addition, in a murine AIDS model that the treatment with purified substance significantly prevented splenomegaly and the enlargement of cervical lymph nodes in C57Bl/6 mice chronically infected with the pool of murine leukemia retroviruses were also reported [173].

### 3.7. Combretum molle (R. Br. ex. G. Don.) Engl & Diels (Combretaceae)

In vitro anti-HIV activity of various extracts prepared from the stem bark of *Combretum molle* widely used in Ethiopian traditional medicine for the treatment of liver diseases, malaria and tuberculosis has been assessed against human imnmuunodeficiencvy virus type 1 (HIV-1) and type 2 (HIV-2). The extracts were prepared by percolation with petroleum ether, chloroform, acetone and the methanol extract was obtained by successive hot extraction using Soxhlet apparatus. Selective inhibition of viral growth was assessed by the simultaneous determination of the in vitro cytotoxicity of each of the extracts against MT-4 cells [375]. The results obtained in this study indicate that the acetone fraction possessed the highest selective inhibition of HIV-1 replication. Phytochemical investigation of the acetone fraction has resulted in the isolation of two tannins and two oleanane-type pentacyclic triterpene glycosides. One of the tannins was identified as punicalagin (an ellagitannin), while the structure of the other (CM-A) has not yet been fully elucidated. On the other hand, both punicalagin and CM-A had displayed selective inhibition of HIV-1 replication with selectivity indices (ratio of 50% cytotoxic concentration to 50% effective antiviral concentration) of 16 and 25, respectively and afforded cell protection of viral induced cytopathic effect of 100% when compared with control samples.

### 3.8. Diospyros lotus L. (Ebenaceae)

Methanol extract of the fruits of *Diospyros lotus* were tested for anti-HIV-1 activity. Gallic acid was found the most active compound against HIV-1 with Therapeutic Index (TI) value of >32.84 and the other compounds were less potent active. *Diospyros lotus* fruits could provide a chemical reservoir of anti-HIV agents. All identified compounds were tested for their cytotoxicity and anti-HIV-1 activities. For positive control, the marketed drug azido-thymidine (AZT) was also tested as a reference according to the same methods. The activity data were described as 50% cytotoxicity concentration (CC_50_), 50% effective concentration (EC_50_%), and therapeutic index (TI), the ratio of CC_50_/EC_50_). Seven isolated phenolic compounds (CC_50_ > 200 μg/mL) have shown less toxicity to C8166 cells compared to ellagic acid (CC_50_ = 35.84 μg/mL). Gallic acid inhibited HIV-1 replication with EC_50_ value of 6.09 μg/mL and TI value of > 32.84, higher than any other compounds. The anti-HIV-1 activity assay was performed by syncytia formation. The seven phenolic compounds showed a good anti-HIV-1 activity and compound gallic acid, a simple tannin compound was the most active and its TI value was the highest [376].

### 3.9. Dittrichia viscosa (L.) Greuter (Asteraceae)

The aqueous extract of *Dittrichia viscosa* was tested for its ability to inhibit the HIV replication. HIV infection of MT-2 cells was used for evaluating antiviral test as rapid and sensitive assay system for the detection of potential antiviral drugs effective against AIDS. The aqueous extract of *Dittrichia viscosa* has showed inhibitory effects against HIV-1 induced infections in MT-2 cells at concentrations ranging from 25 to 400 μg/mL of therapeutic interest [377].

### 3.10. Galanthus nivalis L. (Amaryllidaceae)

Agglutinin isolated from *Galanthus nivalis* (GNA) is a member of a superfamily of strictly mannose-binding specific lectins widespread among monocotyledonous plants, and is well-known to possess a broad range of biological functions such as anti-tumor, anti-viral and anti-fungal activities [378]. The molecular mechanisms of GNA exerting anti-viral activities by blocking the entry of the virus into its target cells, preventing transmission of the virus as well as forcing virus to delete glycan in its envelope protein and triggering neutralizing antibody were discussed. These findings may provide a new perspective of GNA-related lectins as potential drugs for virus therapeutics in the future.

### 3.11. Garcinia edulis Exell (Clusiaceae)

The isoprenylated xanthone derivative determined as 1,4,6-trihydroxy-3-methoxy-2-(3-methyl-2-butenyl)-5-(1,1-dimethyl-prop-2-enyl)xanthone was isolated from the ethanolic extract of the root bark of *Garcinia edulis*. It exhibited anti-HIV-1 protease activity with IC_50_ value of 11.3 μg/mL in vitro while acetyl pepstatin was used as a positive control possessing an anti-HIV-1 PR activity of IC_50_ value of 2.2 μg/mL [379]. However, this compound has also showed potent lethality with LC_50_ value of 2.36 μg/mL against brine shrimp larvae in vitro.

### 3.12. Helichrysum populifolium (Asteraceae)

The methanol:water (1:1) extract of the aerial parts of *Helichrysum populifolium* growing in South Africa was tested for the anti-HIV test by using HeLa-SXR5 expressed the CD4 receptor and the CXCR4/CCR5 chemokine receptors and the extract was found to be active (IC_50_ value of 12 μg/mL) [123]. The anti-HIV compounds identified from *H. populifolium* were three dicaffeoylquinic acid derivatives, i.e., 3,4-dicaffeoylquinic acid, 3,5-dicaffeoylquinic acid and 4,5-dicaffeoylquinic acid as well as two tricaffeoylquinic acid derivatives, i.e., 1,3,5-tricaffeoylquinic acid and either 5-malonyl-1,3,4-tricaffeoylquinic or 3-malonyl-1,4,5-tricaffeoylquinic acid.

### 3.13. Hoodia gordonii (Masson) Sweet ex Decne (Apocynaceae)

The in vitro anti-HIV potential of the ethanol and ethylacetate extracts of *Hoodia gordonii* was examined. Both extracts had shown good inhibition in a dose-dependent manner against HIV-1 reverse transcriptase (RT) with IC_50_ values of 73.55 ± 0.04 and 69.81 ± 9.45 μg/mL, respectively. Doxorubicin, a known RT inhibitor was used as a positive control and inhibited HIV RT by 68% at 25 μg/mL (IC_50_ < 25 μg/mL). Both extracts also demonstrated inhibitory activity against HIV protease (PR) with IC_50_ values of 97.29 ± 0.01 and 63.76 ± 9.01 μg/mL for ethanol and ethyl acetate extracts, respectively. Acetyl pepstatin was used as a known PR inhibitor and inhibited HIV PR by as much as 82% at 50 μg/mL (IC_50_ < 50 μg/mL). In addition, both ethanol and ethyl acetate extracts had weak inhibition against HIV-1 integrase (IN) with <50% inhibition at the highest concentration tested of 400 μg/mL. Sodium azide was used as a positive control compound for IN inhibition [101]. In the same study, phytochemical screening of *Hoodia gordonii* was revealed the presence of phenolics, alkaloids, terpenes, steroids, cardiac glycosides and tannins in the ethanolic extract, while the ethyl acetate extract only showed the presence of phenolics, cardiac glycosides and steroids.

### 3.14. Hypericum perforatum L. (Hypericaceae)

*Hypericum perforatum*, known as St. John’s Wort, has been used for medicinal purposes, particularly wound healing, since the Middle Ages. It was also used in treatment of AIDS [380]. In a clinical trial, hypericin and pseudohypericin isolated from this plant have shown antiretroviral activity in HIV-infected patients [381].

### 3.15. Hyssopus officinalis L. (Lamiaceae)

*Hyssopus officinalis* has been used as herbal medicine and the extracts of this species have demonstrated strong activity against HIV-1 due to the content of polysaccharide-type compounds [252]. The 50% hydroalcoholic extract of *Hysoppus officinalis* was examined for its ability to inhibit HIV replication. Among the variety of assays for evaluating antiviral tests, HIV infection of MT-2 cells was used as a rapid and sensitive assay system for the detection of potential antiviral drugs effective against AIDS. This extract had shown inhibitory effects against HIV-1 induced infections in MT-2 cells at concentrations ranging from 50 to 100 μg/mL.

### 3.16. Justicia gendarussa Burm. f. (syn: Gendarussa vulgaris Nees) (Acanthaceae)

*Justicia gendarussa* was identified as a potent anti-HIV-1 active lead from the evaluation of over 4500 plant species growing in Vietnam and Laos by showing complete inhibition against HIV replication at a concentration 20 μg/mL. The methanol extract of the stems and barks of the plant have led to the isolation of justiprocumins A and B as new arylnaphthalide lignan glycosides by using bioassay-guided isolation. Justiprocumin B has shown potent activity against a broad spectrum of HIV strains with IC_50_ values in the range of 15–21 nM (AZT, IC_50_ 77–95 nM, as positive control). Justiprocumin B also displayed potent inhibitory activity against the NRTI (nucleoside reverse transcriptase inhibitor)-resistant isolate (HIV-1_1617-1_) of the analogue (AZT) as well as the NNRTI (non-nucleoside reverse transcriptase inhibitor)-resistant isolate (HIV-1_N119_) of the analogue (nevaripine) [382]. The dichloromethane plant extract has shown complete inhibition of HIV replication at a concentration of 20 μg/mL. This bioactivity was confirmed by the evaluation of the MeOH extract prepared from a re-collected sample of the same plant, with HIV-1 replication inhibition at an IC_50_ value of 40 ng/mL. Bioassay-guided separation of the extracts of the stems and roots of this plant led to the isolation of an anti-HIV arylnaphthalene lignan (ANL) glycoside, patentiflorin A. Evaluation of the compound against both the M- and T-tropic HIV-1 isolates showed it to possess a significantly higher inhibition effect than the clinically used anti-HIV drugs known as the nucleotide analogue (AZT) and non-nucleotide analogue (nevaripine). Thus, patentiflorin A has the potential to be developed as a novel anti-HIV drug [382]. Patentiflorin A showed anti-HIV-1 activity with an IC_50_ value of 26.9 nM in the defective HIV-based pseudotyped assay. The results clearly showed that patentiflorin A has broad-spectrum activity against both M-tropic and T-tropic HIV-1 isolates with IC_50_ values lower than that of AZT, the first anti-HIV drug developed and still used in the treatment of HIV/AIDS. Like AZT, it inhibited the particle production of all four HIV-1 isolates effectively in a dose-dependent manner. Patentiflorin A gave an IC_50_ value of 24–37 nM, compared to 77–95 nM for AZT.

### 3.17. Momordica charantia L. (Cucurbitacae)

*Momordica charantia*, known as bitter melon and widely exploited in folkloric medicine, has been shown to inhibit HIV-1 reverse transcriptase due to its protein coded as MRK29 [383]. The efficacies and molecular mechanisms of bitter gourd-induced anti-diabetic, anti-HIV, and antitumor activities contributed by over twenty active components were determined. Therefore, bitter gourd is a cornucopia of health and it has been deserved in-depth investigations for clinical application in the future.

Anti-HIV properties of the fruit pulp extract of *Momordica balsamina*, commonly used in the northern part of Nigeria for its anti-viral efficacy in poultry, was studied in vitro and was found as a potent inhibitor of HIV-1 replication; further research on fruit pulp extract should be pursued for its potential in the prophylaxis and therapy of retroviral infections in humans [384].

### 3.18. Pachyma hoelen Rumph (Polyporaceae)

The hexane extract of *Pachyma hoelen* Rumph used in folk medicine in Korea was shown to have the best anti-HIV-1 activity compared to the other extracts tested. This extract had 37.3 μg/mL (EC_50_) on the p24 antigen assay as the highest value, 36.8% on the RT activity test (at 200 μg/mL). In addition, this extract had shown protective effects on infected MT-4 cells; the protection was the highest observed at 58.2%. The 50% cytotoxic concentration (CC_50_) of the hexane extract of this plant species was found 100.6 μg/mL [196].

### 3.19. Phyllanthus pulcher (Euphorbiaceae)

The methanol extract of *Phyllanthus* species growing in Malaysia was evaluated for anti-HIV-1 reverse transcriptase (RT) activity using the HIV-RT assay by inhibition of the HIV-1 RT enzyme based on their IC_50_ values. Azido-deoxythymidine-triphosphate (AZT151TP) was used as a positive control. The inhibition of HIV-RT for *P. pulcher* was IC_50_ of 5.9 μg/mL [385].

### 3.20. Rhus chinensis Mill (Anacardiaceae)

The anti-HIV-1 activities of the petroleum ether, ethyl acetate, butanol and aqueous extracts of *Rhus chinensis* growing in China and Japan where it is known as Chinese Sumac were examined. The petroleum ether extract had significantly suppressed HIV-1 activity in vitro and was found to inhibit syncytium formation and HIV-1 p24 antigen at non-cytotoxic concentrations, the EC_50_ were 0.71 and 0.93 μg/mL respectively. The petroleum ether extract had no activity on inhibiting HIV-1 recombinant RT or HIV-1 entry into host cells cycle. *R. chinensis* would be a useful medicinal plant for the chemotherapy of HIV-1 infection. The petroleum ether extract of this plant likely inhibit the post entry steps or target the new sites of HIV-1 replication [386].

### 3.21. Sceletium tortuosum (L.) N.E. Brown (Aizoaceae)

The ethanolic and ethyl acetate extracts prepared from the whole part of *Sceletium tortuosum*, distributed throughout southern Africa, were investigated for their inhibitory activity against HIV-1 enzymes including protease (PR), reverse transcriptase (RT) and integrase (IN) [172]. The HIV-1 RT inhibition testing had IC_50_ values of <50 and 121.7 ± 2.5 μg/mL for ethanol and ethyl acetate extracts, respectively. In addition, both extracts had also inhibited HIV-1 PR with IC_50_ values < 100 μg/mL. *Sceletium tortuosum* might be a potential source of new lead compounds in the development of new anti-HIV compounds [67].

### 3.22. Smilax corbularia Kunth (Smilaceae)

The ethanolic and aqueous extracts were tested for their inhibitory effects against HIV-1 protease (HIV-PR) and HIV-1 integrase (HIV-1 IN). The results indicated that the ethanolic extract of *S. corbularia* exhibited anti-HIV-1 IN activity with an IC_50_ value of 1.9 μg/mL, approximately two-fold lower than that of suramin (IC_50_ = 3.4 μg/mL) as the positive control. The value of IC_50_ = 5.4 μg/mL was determined for the water extract of *Smilax corbularia* [120].

### 3.23. Terminalia paniculata (Combretaceae)

The in vitro anti-HIV1 activity of acetone and methanol extracts prepared from the fruits of *Terminalia paniculata* was examined. The EC_50_ values of the acetone and methanol extracts of *T. paniculata* were ≤10.3 μg/mL. The enzymatic assays were performed to determine the mechanism of action and indicated that the anti-HIV1 activity might be due to inhibition of reverse transcriptase (≥77.7% inhibition) and protease (≥69.9% inhibition) enzymes [387].

### 3.24. Tuberaria lignosa (Sweet) Sampaio (Asteraceae)

*Tuberaria lignosa* was widely used in the folk medicine to treat diseases of viral origin of the Iberian Peninsula and the ethanolic and aqueous extracts were evaluated for its anti-HIV activity by inhibiting HIV replication. The toxicity of the extracts to MT-2 cells was also investigated. The ethanolic extract was especially toxic, which prevented the evaluation of their potential antiviral effects at higher concentrations. However, the aqueous extract of *T. lignosa* tested was relatively nontoxic to human lymphocytic MT-2 cells, but did show anti-HIV activity at concentrations ranging from 12.5 to 50 μg/mL [61].

In conclusion, terrestrial plants produce secondary metabolites for their chemical defense, which possess unique chemical structures and have played pivotal roles in human health. There is continuous need to introduce new drug candidates to treat diseases and the drug discovery process can be realized using both ancient and modern research methodologies in a complementary manner. Some medicinal plants are still unexplored; therefore there are numerous avenues of research for the determination of their biological activities. In this review, the anti-HIV activity of some plant extracts and their potential utilization for anti-HIV agents have been summarized. Among them *Calendula officinalis, Justicia gendarussa* and *Sceletium tortuosum* might be useful potential sources for new lead compounds in the development of new candidates with anti-HIV properties of therapeutic interest. These studies are considered to be one of the most important approaches toward effective therapy for AIDS.

## 4. Human Clinical Trials

There are few reports about using the herbal medicine in clinical studies and treatment for HIV/AIDS. This area is not well researched. But, in Africa, where HIV, AIDS and HIV related diseases are the most widespread problems, herbal medicines are used as primary treatment for them. Highly active antiretroviral therapy is also applied in China and implies three types of treatment systems. One of them is traditional Chinese medicine provided by trained Chinese herbalists. There are several randomized studies related to beneficial effects of traditional medical plants on patients with HIV or AIDS which were compared with control group (without treatment and placebo). The effects in promoting CD4^+^ cells were followed. Based on selected, different, studies approximately eleven different Chinese traditional medical plants such as *Panax ginseng*, *Astragalus membranaceus*, *Lycium barbarum*, *Trichosanthis kirilowii*, and *Viola mandshurica* were tested in about 1000 patients within different studies. Compared with placebo, treatment with traditional medical plants showed positive effect, increasing CD4 cells, but studies need to be improved [388].

Some Chinese herbal preparation which consists of 14 plants (*Coptis chinensis*, *Jasminum officinale*, *Wolfiporia extensa*, *Sparganium stoloniferum*, *Polygonatum odoratum*, and *Scrophularia buergeriana* was investigated during 24 weeks and observed to have increased plasma CD4 count and also showed inhibition of HIV growth [389]. According to one US study, 26% of HIV-infected people use herbal medicine as part of their treatment. A European study showed that herbal medicines are used by approximately 25% of HIV infected people [390].

The study, which included 366 HIV-positive African-American women who were enrolled in herbal medicine therapy, showed that in these patients experienced 1.69 time stronger anti-retroviral effect compered to women not using the therapy based on medical plants [391]. Thirty-three HIV-positive volunteers (7 men and 26 women between 22 and 43 years of age) who used *Calendula officinalis* or *Agastache rugosa* were evaluated in South Africa. There was a significant decrease in viral loads and in CD4 T-cell counts [392].

The Ministry of Health of South Africa is actively promoting the use of traditional medicines with antiretroviral treatments and recommended two plants remedies which have been used for HIV/AIDS treatment: *Hypoxis hemerocallidea* and *Sutherlandia frutescens* [393]. Also, in Romania it was noticed that children with AIDS who were treated with natural herbal remedies showed a decrease in mortality rate [393]. Furthermore, in blood samples of 30 adults who used an extract of *Alternanthera pungens*, a significant increase of CD4 and CD8 lymphocytes was observed [394].

The study which was conducted to demonstrate using medical plants in different districts in Uganda, where this disease first described and one million habitants are infected, 25 traditional medicine practitioners were interviewed. The practitioners received on average 29 (range, 2–250) patients each year. They mentioned 145 belong to families Asteraceae, Fabaceae and Euphorbiaceae. It was also noted that the most used plants were *Aloe* spp., *Erythrina abyssinica*, *Sarcocephalus latifolius*, *Psorospermum febrifugum*, *Mangifera indica*, and *Warburgia salutaris*. In patients involved in herbal medicine treatment progressive loss of CD4 positive T-cell lymphocytes in the blood was observed [311].

## 5. Conclusions

Focusing on phytochemicals that have reached clinical trials, if there are any; highlighting medicinal plants where high level of scientific evidence has been reached; future perspectives.

Although there have been major accomplishments in HIV chemotherapy, there remains a need for new anti-HIV drug discovery, and medicinal plants can play an important role in this endeavor. Several plant species have shown remarkable anti-HIV activity, especially *Artemisia annua*, *Garcinia edulis*, *Justicia gendarussa*, *Phyllanthus pulcher*, *Rhus chinensis*, *Smilax corbularia*, *Terminalia paniculata*, and *Tuberaria lignosa*. These plant species are worthy of further study for the development of new anti-HIV chemotherapeutic options. In particular, in vivo testing and, ultimately, human clinical trials need to be carried out on key lead plants and phytochemical isolates. In addition, continuous evaluation of medicinal plants for anti-HIV activity should be pursued.

## Figures and Tables

**Table 1:** List of plant species exhibiting different human immunodeficiency virus (HIV)-inhibition activities.

Family	Plant	Plant Part	HIV-RT	HIV-PR	HIV-IN	Anti-HIV
Acanthaceae	*Andrographis paniculata* (Burm. f.) Wall. ex Nees	Aerial part				Crude [51,52]
Acanthaceae	*Avicennia marina* var. *rumphiana* (Hallier f.) Bakh.	Seed				Iridoid glycoside [53]
Acanthaceae	*Avicennia officinalis* L.	Leaf	Crude [54]	Crude [55]		
Acanthaceae	*Justicia adhatoda* L.					Crude [56]
Acanthaceae	*Justicia gendarussa* Burm.f.	Aerial part	Crude [57]			
Acanthaceae	*Rhinacanthus nasutus* (L.) Kurz	Aerial part	Crude [58]			Crude [59]
Acanthaceae	*Strobilanthes cusia* (Nees) Kuntze					Crude [60]
Acoraceae	*Acorus calamus* L.	Rhizome	Crude [58]			
Adoxaceae	*Sambucus ebulus* L.	Whole plant				Crude [61]
Adoxaceae	*Sambucus nigra* L.	Whole plant	Crude [62]			Crude [61,63]
Adoxaceae	*Sambucus racemosa* L.	Leaf, Fruit	Crude [62,64]			
Adoxaceae	*Sambucus williamsii* Hance	Roots , Fruits				Crude [65,66]
Adoxaceae	*Viburnum opulus* L.	Leaf, Fruit	Crude [62]			
Aizoaceae	*Sceletium tortuosum* (L.) N.E. Br.		Crude [67]	Crude [67]	Crude [67]	
Alismataceae	*Alisma plantago-aquatica* L.	Rhizome		Crude [68]		
Amaranthaceae	*Achyranthes bidentata* Blume					Crude [66,69]
Amaranthaceae	*Achyranthes japonica* (Miq.) Nakai	Root				Crude [66]
Amaranthaceae	*Aerva lanata* (L.) Juss. ex Schult.	Root	Phytotesrols [70]			
Amaranthaceae	*Alternanthera brasiliana* (L.) Kuntze					Crude [71]
Amaranthaceae	*Alternanthera philoxeroides* (Mart.) Griseb.	Aerial part				Crude [72,73]
Amaryllidaceae	*Allium sativum* L.	Bulb	Crude [58]			Crude [56]
Amaryllidaceae	*Crinum amabile* Donn ex Ker Gawl.	Bulb	Crude [74]			
Amaryllidaceae	*Crinum macowanii* Baker	Bulb	Crude [75]	Crude [75]		
Amaryllidaceae	*Haemanthus albiflos* Jacq.					Crude [76]
Amaryllidaceae	*Leucojum vernum* L.	Bulb	Alkaloids [77]			
Amaryllidaceae	*Pamianthe peruviana* Anonymous	Bulb	Crude [74]			
Amaryllidaceae	*Tulbaghia alliacea* L. f.	Bulb				Crude [78]
Amaryllidaceae	*Tulbaghia violacea* Harv.	Bulb	Crude [75]	Crude [75]		
Anacardiaceae	*Lannea edulis* (Sond.) Engl.	Bulb				Crude [79]
Anacardiaceae	*Mangifera indica* L.	Stem bark				Crude [80]
Anacardiaceae	*Rhus chinensis* Mill.	Leaf, Root, Stem, Bark, Fruit				Read phyto [81]
Anacardiaceae	*Schinus molle* L.	Leaf				Crude [82]
Anacardiaceae	*Spondias pinnata* (L. f.) Kurz	Fruit	Crude [58]			
Anacardiaceae	*Toxicodendron acuminatum* (DC.) C.Y. Wu & T.L. Ming	Gall		Crude [83]		
Ancistrocladaceae	*Ancistrocladus korupensis* D.W. Thomas & Gereau	Root	Naphthylisoquinoline alkaloids [84]			Crude [85] Naphthylisoquinoline alkaloids [86]
Annonaceae	*Annona glabra* L.	Fruit				Alkaloids [87]
Annonaceae	*Annona senegalensis* Pers.	Leaf				Crude [80]
Annonaceae	*Annona squamosa* L.	Fruit				Diterpenoids [88]
Annonaceae	*Dasymaschalon rostratum* Merr. & Chun	Stem	Phenylpropanoid derivatives [89]			
Annonaceae	*Dasymaschalon sootepense* Craib	Leaf	Alkaloids, Flavonoid [90]			
Annonaceae	*Polyalthia suberosa* (Roxb.) Thwaites	Stem bark	Crude [57]			Triterpene [91] and 2-substituted furans [92]
Annonaceae	*Xylopia frutescens* Aubl.	Bark		Crude [93]		
Apiaceae	*Alepidea amatymbica* Eckl. & Zeyh.					Rosmarinic acid [94]
Apiaceae	*Ammi visnaga* (L.) Lam.	Fruit		Crude [95]		
Apiaceae	*Anethum graveolens* L.	Seed		Crude [83]		
Apiaceae	*Angelica dahurica* (Fisch.) Benth. & Hook. f.	Root				Crude [66]
Apiaceae	*Angelica grosseserrata* Maxim.	Aerial part		Crude [96]		
Apiaceae	*Apium graveolens* L.	Fruit		Crude [83]		
Apiaceae	*Cryptotaenia japonica* Hassk.	Aerial part		Crude [96]		
Apiaceae	*Foeniculum vulgare* Mill.	Fruit				Crude [66]
Apiaceae	*Lomatium suksdorfii* (S. Watson) J.M. Coult. & Rose	Fruit				Coumarin [97]
Apiaceae	*Mulinum ulicinum* Gillet & Hook.	Leaf, Stem				Crude [82]
Apiaceae	*Ridolfia segetum* (L.) Moris		Essential oils [98]			
Apiaceae	*Saposhnikovia divaricate* (Turcz.) Schischk.			Crude [60,68]		Crude [66]
Apiaceae	*Torilis japonica* (Houtt.) DC.	Seed		Crude [96]		
Apocynaceae	*Alstonia scholaris* (L.) R. Br.	Stem bark				Crude [56]
Apocynaceae	*Carissa bispinosa* Desf. ex Brenan	Roots				Crude [99]
Apocynaceae	*Catharanthus roseus* (L.) G. Don	Leaf				Crude [56]
Apocynaceae	*Cynanchum atratum* Bunge	Root				Crude [66]
Apocynaceae	*Cynanchum paniculatum* (Bunge) Kitag.	Root				Crude [66]
Apocynaceae	*Gymnema sylvestre* (Retz.) R. Br. ex Schult.			Crude [99]		
Apocynaceae	*Hemidesmus indicus* (L.) R. Br. ex Schult.		Crude [100]			
Apocynaceae	*Hoodia gordonii* (Masson) Sweet ex Decne.		Crude [101]	Crude [101]	Crude [101]	
Apocynaceae	*Parameria laevigata* (Juss.) Moldenke	Bark		Crude [68]		
Apocynaceae	*Rauvolfia serpentine* (L.) Benth. ex Kurz					Crude [56]
Apocynaceae	*Solenostemma argel* (Delile) Hayne	Root		Crude [95]		
Apocynaceae	*Tabernaemontana stapfiana* Britten		Crude [102]			
Araceae	*Alocasia odora* (Roxb.) K. Koch	Rhizome		Crude [68]		
Araliaceae	*Acanthopanax koreanum* Nakai	Stem bark		Crude [96]		Crude [96]
Araliaceae	*Eleutherococcus sessiliflorus* (Rupr. & Maxim.) S.Y. Hu					Crude [66]
Araliaceae	*Kalopanax pictus* (Thunb.) Nakai	Stem bark				Crude [66]
Araliaceae	*Panax ginseng* C.A. Mey.	Root		Triterpenoids [103]		Saponin [104]
Araliaceae	*Panax notoginseng* (Burkill) F.H. Chen ex C.H. Chow			Crude [60]	Crude [105]	
Araliaceae	*Panax zingiberensis* C.Y. Wu & K.M. Feng	Rhizome				Zingibroside [106]
Arecaceae	*Areca catechu* L.	Seed		Crude [60,83]		
Arecaceae	*Attalea tessmannii* Burret	Seed				Crude [82]
Aristolochiaceae	*Aristolochia bracteolate* Lam.	Fruit	Crude [74]	Crude [95]		
Aristolochiaceae	*Aristolochia contorta* Bunge	Fruit				Crude [66]
Aristolochiaceae	*Aristolochia manshuriensis* Kom.	Stem				Oxoperezinone [107]
Aristolochiaceae	*Asarum sieboldii* Miq.	Root				Crude [66]
Asparagaceae	*Anemarrhena asphodeloides* Bunge	Rhizome		Crude [68]		
Asparagaceae	*Asparagus cochinchinensis* (Lour.) Merr.	Root				Crude [66]
Asparagaceae	*Asparagus racemosus* Willd.	Root				Crude [56]
Asparagaceae	*Dracaena cochinchinensis* (Lour.) S.C. Chen		Crude [58]			
Asteraceae	*Acanthospermum hispidum* DC.	Aerial part	Crude [74]			
Asteraceae	*Achyrocline alata* (Kunth) DC.	Flower, Stem				Crude [82]
Asteraceae	*Achyrocline flaccida* (Weinm.) DC.					Crude [108]
Asteraceae	*Achyrocline satureioides* (Lam.) DC.	Flower				Crude [82]
Asteraceae	*Ainsliaea acerifolia* Sch. Bip.	Whole plant		Crude [96]		
Asteraceae	*Ambrosia artemisiifolia* L.	Whole plant		Crude [96]		
Asteraceae	*Ambrosia maritima* L.	Aerial part		Crude [95]		
Asteraceae	*Ambrosia peruviana* All.	Leaf, stem				Crude [82]
Asteraceae	*Anvillea garcinii* (Burm. f.) DC.	Aerial part				Germacranolides [109]
Asteraceae	*Arctium lappa* L.	Aerial part		Crude [60]	Crude [105]	Crude [51,66,72]
Asteraceae	*Artemisia absinthium* L.	Leaf				Crude [82]
Asteraceae	*Artemisia annua* L.	Aerial part				Crude [66]
Asteraceae	*Artemisia capillaris* Thunb.	Aerial part, Seed		Crude [68]		Crude [66]
Asteraceae	*Artemisia princeps* Pamp.	Leaf		Crude [68,96]		
Asteraceae	*Artemisia verlotorum* Lamotte					Crude [110]
Asteraceae	*Aspilia pluriseta* Schweinf. ex Schweinf.					Crude [111]
Asteraceae	*Aster tataricus* L. f.	Root		Crude [68]		
Asteraceae	*Atractylodes japonica* Koidz.	Root		Crude [96]		Crude [66]
Asteraceae	*Atractylodes lancea* (Thunb.) DC.	Rhizome		Crude [68]		Crude [112]
Asteraceae	*Atractylodes ovate* (Thunb.) DC.	Rhizome		Crude [68]		
Asteraceae	*Baccharis genistelloides* (Lam.) Pers.	Leaf, stem				Crude [82]
Asteraceae	*Baccharis latifolia* (Ruiz & Pav.) Pers.	Leaf, stem				Crude [82]
Asteraceae	*Baccharis trimera* (Less.) DC.	Leaf, stem				Crude [82]
Asteraceae	*Baccharis trinervis* Pers.	Aerial part				Crude [93]
Asteraceae	*Bidens pilosa* L.	Aerial part				Crude [93]
Asteraceae	*Blumea balsamifera* (L.) DC.				Crude [113]	Crude [113]
Asteraceae	*Breea segeta* (Bunge) Kitam.	Aerial part				Crude [66]
Asteraceae	*Calea jamaicensis* (L.) L.	Root				Crude [93]
Asteraceae	*Calendula officinalis* L.	Leaf	Crude [114]			Crude [115]
Asteraceae	*Carlina acaulis* L.	Leaf	Crude [62]			
Asteraceae	*Carpesium abrotanoides* L.			Crude [96]		
Asteraceae	*Carthamus tinctorius* L.	Flower				Crude [66]
Asteraceae	*Centratherum punctatum* Cass.	Leaf	Crude [114]			
Asteraceae	*Chrysanthemum indicum* L.	Capitulum		Crude [60]	Crude [105]	
Asteraceae	*Chrysanthemum morifolium* Ramat.	Capitulum	Flavonoids [116]	Crude [60,68]	Crude [105] Flavonoid [117]	Crude [117,118]
Asteraceae	*Cirsium japonicum* DC.			Crude [96]		
Asteraceae	*Eclipta prostrate* (L.) L.	Whole plant		Lactone [119]	Crude [120] Lactone [119]	
Asteraceae	*Elephantopus scaber* L.	Leaf		Crude [68]		
Asteraceae	*Eupatorium lindleyanum* DC.	Aerial part		Crude [96]		
Asteraceae	*Francoeuria crispa* (Forssk.) Cass.					Crude [121]
Asteraceae	*Franseria artemisioides* Willd.	Leaf, stem				Crude [82]
Asteraceae	*Gamochaeta simplicicaulis* (Willd. ex Spreng.) Cabrera		Crude [122]			Crude [108]
Asteraceae	*Geigeria alata* (DC.) Oliv. & Hiern					Crude [121]
Asteraceae	*Gnaphalium sylvaticum* L.	Leaf	Crude [62]			
Asteraceae	*Gynura pseudochina* (L.) DC.	Leaf	Crude [57]			
Asteraceae	*Helianthus tuberosus* L.	Whole plant		Crude [96]		
Asteraceae	*Helichrysum acutatum* DC.	Aerial part				Crude [123]
Asteraceae	*Helichrysum allioides* Less.	Aerial part				Crude [123]
Asteraceae	*Helichrysum anomalum* Less.	Aerial part				Crude [123]
Asteraceae	*Helichrysum appendiculatum* (L. f.) Less.	Aerial part				Crude [123]
Asteraceae	*Helichrysum auronitens* Sch. Bip.	Aerial part				Crude [123]
Asteraceae	*Helichrysum cephaloideum* DC.	Aerial part				Crude [123]
Asteraceae	*Helichrysum chionosphaerum* DC.	Aerial part				Crude [123]
Asteraceae	*Helichrysum confertum* N.E. Br.	Aerial part				Crude [123]
Asteraceae	*Helichrysum cymosum* (L.) D. Don ex G. Don	Aerial part				Crude [123]
Asteraceae	*Helichrysum difficile* Hilliard	Aerial part				Crude [123]
Asteraceae	*Helichrysum drakensbergense* Killick	Aerial part				Crude [123]
Asteraceae	*Helichrysum herbaceum* (Andrews) Sweet	Aerial part				Crude [123]
Asteraceae	*Helichrysum melanacme* DC.	Aerial part				Crude [123]
Asteraceae	*Helichrysum miconiifolium* DC.	Aerial part				Crude [123]
Asteraceae	*Helichrysum natalitium* DC.	Aerial part				Crude [123]
Asteraceae	*Helichrysum nudifolium* (L.) Less.	Aerial part				Crude [123]
Asteraceae	*Helichrysum odoratissimum* (L.) Sweet	Aerial part				Crude [123]
Asteraceae	*Helichrysum oreophilum* Dinter	Aerial part				Crude [123]
Asteraceae	*Helichrysum oxyphyllum* DC.	Aerial part				Crude [123]
Asteraceae	*Helichrysum pallidum* DC.	Aerial part				Crude [123]
Asteraceae	*Helichrysum panduratum* O. Hoffm.	Aerial part				Crude [123]
Asteraceae	*Helichrysum pannosum* DC.	Aerial part				Crude [123]
Asteraceae	*Helichrysum pilosellum* (L. f.) Less.	Aerial part				Crude [123]
Asteraceae	*Helichrysum populifolium* DC.	Aerial part				Crude [123]
Asteraceae	*Helichrysum rugulosum* Less.	Aerial part				Crude [123]
Asteraceae	*Helichrysum splendidum* (Thunb.) Less.	Aerial part				Crude [123]
Asteraceae	*Helichrysum subluteum* Burtt Davy	Aerial part				Crude [123]
Asteraceae	*Helichrysum sutherlandii* Harv.	Aerial part				Crude [123]
Asteraceae	*Helichrysum umbraculigerum* Less.	Aerial part				Crude [123]
Asteraceae	*Helichrysum vernum* Hilliard	Aerial part				Crude [123]
Asteraceae	*Hieracium pilosella* L.	Whole plant				Crude [61]
Asteraceae	*Hieracium umbellatum* L.	Whole plant		Crude [96]		
Asteraceae	*Inula britannica* L.	Flower				Crude [66]
Asteraceae	*Inula helenium* L.	Root				Crude [66]
Asteraceae	*Ixeris tamagawaensis* (Makino) Kitam.	Aerial part				Crude [124]
Asteraceae	*Lactuca raddeana* Maxim.	Whole plant		Crude [96]		
Asteraceae	*Miyamayomena koraiensis* (Nakai) Kitam.	Root		Crude [96]		
Asteraceae	*Mutisia acuminata* Ruiz & Pav.	Leaf				Crude [82]
Asteraceae	*Perezia multiflora* (Bonpl.) Less.	Leaf				Crude [82]
Asteraceae	*Pilosella officinarum* F.W. Schultz & Sch. Bip.	Whole plant				Crude [61]
Asteraceae	*Psiadia dentata* (Cass.) DC.					Coumarin [125]
Asteraceae	*Santolina oblongifolia* Boiss.	Whole plant				Crude [61]
Asteraceae	*Saussurea seoulensis* Nakai	Whole plant		Crude [96]		
Asteraceae	*Schkuhria pinnata* (Lam.) Kuntze ex Thell.	Leaf				Crude [82]
Asteraceae	*Senecio comosus* Sch. Bip.	Leaf				Crude [82]
Asteraceae	*Senecio mathewsii* Wedd.	Leaf				Crude [82]
Asteraceae	*Senecio rhizomatus* Rusby	Leaf				Crude [82]
Asteraceae	*Senecio scandens* Buch.-Ham. ex D. Don	Whole plant		Crude [60]	Crude [105]	Crude [72]
Asteraceae	*Serratula coronate* L.	Aerial part		Crude [96]		
Asteraceae	*Sigesbeckia glabrescens* (Makino) Makino	Whole plant				Crude [66]
Asteraceae	*Sonchus oleraceus* L.	Leaf				Crude [82]
Asteraceae	*Symphyotrichum undulatum* (L.) G.L.Nesom	Aerial part			Quinic acid [126]	
Asteraceae	*Tagetes riojana* M. Ferraro	Leaf				Crude [82]
Asteraceae	*Tanacetum microphyllum* DC.	Whole plant				Crude [61]
Asteraceae	*Taraxacum mongolicum* Hand.-Mazz.	Whole plant		Crude [68]		
Asteraceae	*Xanthium spinosum* L.	Flower				Crude [82]
Berberidaceae	*Berberis holstii* Engl.	Root and Leaf				Crude [127]
Berberidaceae	*Epimedium grandiflorum* C. Morren	Aerial part				Crude [21,72]
Berberidaceae	*Epimedium sagittatum* (Siebold & Zucc.) Maxim.	Leaf		Crude [68]		
Berberidaceae	*Nandina domestica* Thunb.	Leaf		Crude [68]		
Betulaceae	*Alnus firma* Siebold & Zucc.	Leaf	Triterpenoids [128]			
Betulaceae	*Alnus incana* (L.) Moench	Leaf	Crude [62]			
Bignoniaceae	*Kigelia Africana* (Lam.) Benth.	Fruit	Crude [102]			
Bignoniaceae	*Spathodea campanulata* P. Beauv.	Stem bark				Crude [129]
Bignoniaceae	*Tecomella undulata* (Sm.) Seem.	Aerial part				Crude [130]
Blechnaceae	*Blechnum spicant* (L.) Sm.	Leaf	Crude [62]			
Blechnaceae	*Brainea insignis* (Hook.) J. Sm.	Rhizome		Crude [68]		
Blechnaceae	*Woodwardia orientalis* Sw.	Rhizome		Crude [68]		
Blechnaceae	*Woodwardia unigemmata* (Makino) Nakai	Rhizome		Crude [60]	Crude [105]	Crude [72]
Boraginaceae	*Brachybotrys paridiformis* Maxim. ex Oliv.	Leaf		Crude [96]		
Boraginaceae	*Cordia spinescens* L.	Leaf		Crude [93]	Crude [93]	
Boraginaceae	*Lithospermum erythrorhizon* Siebold & Zucc.	Root		Crude [60,68]	Crude [105]	Crude [72,131]
Boraginaceae	*Lobostemon trigonus* H. Buek		Crude [132]			
Brassicaceae	*Brassica juncea* (L.) Czern.	Semen	Crude [133]			Crude [66]
Brassicaceae	*Brassica oleracea* L.		Crude [134]			
Brassicaceae	*Brassica rapa* L.		Crude [134]			
Brassicaceae	*Capsella bursa-pastoris* (L.) Medik.	Whole plant				Crude [82]
Brassicaceae	*Lepidium abrotanifolium* Turcz.	Leaf				Crude [82]
Brassicaceae	*Raphanus raphanistrum* L.					Crude Inhibition [66]
Cactaceae	*Pereskia bleo* (Kunth) DC.	Whole plant				Crude [93]
Calophyllaceae	*Marila pluricostata* Standl. & L.O. Williams					Phenylcoumarins [135]
Campanulaceae	*Adenophora triphylla* (Thunb.) A. DC.	Root				Crude [66]
Campanulaceae	*Platycodon grandiflorus* (Jacq.) A. DC.	Root		Crude [68]		
Cannabinaceae	*Cannabis sativa* L.	Fruit		Crude [68]		
Cannabinaceae	*Humulus lupulus* L.					Flavonoid [136]
Cannaceae	*Canna indica* L.	Rhizome	Crude [57]			
Canellaceae	*Warburgia ugandensis* Sprague		Crude [102]			
Capparaceae	*Boscia senegalensis* (Pers.) Lam. ex Poir.	Leaf	Crude [74]			
Capparaceae	*Capparis decidua* (Forssk.) Edgew.	Stem	Crude [74]			
Capparaceae	*Crateva religiosa* G. Forst.	Bark		Crude [83]		
Caprifoliaceae	*Lonicera japonica* Thunb.	Flower bud	Crude [137]	Crude [60,68]	Crude [105]	Crude [66,72]
Caprifoliaceae	*Patrinia scabiosifolia* Link	Root		Crude [96]		Crude [66]
Caprifoliaceae	*Patrinia villosa* (Thunb.) Dufr.	Root		Crude [68,96]		
Caprifoliaceae	*Valeriana coarctata* Ruiz & Pav.	Leaf				Crude [82]
Caprifoliaceae	*Valeriana micropterina* Wedd.					Crude [82]
Caprifoliaceae	*Valeriana thalictroides* Graebn.	Root				Crude [82]
Caprifoliaceae	*Weigela subsessilis* L.H. Bailey	Stem		Crude [96]		
Caryophyllaceae	*Drymaria cordata* (L.) Willd. ex Schult.	Leaf				Crude [138]
Caryophyllaceae	*Drymaria diandra* Blume					Alkaloid [139]
Caryophyllaceae	*Silene seoulensis* Nakai	Aerial part		Crude [96]		
Celastraceae	*Cassine crocea* (Thunb.) C.Presl					Glycoside [140]
Celastraceae	*Cassine schlechteriana* Loes.					Crude [141]
Celastraceae	*Celastrus hindsii* Benth.					triterpene [142]
Celastraceae	*Celastrus orbiculatus* Thunb.	Root		Crude [96]		Crude [143]
Celastraceae	*Euonymus alatus* (Thunb.) Siebold	Leaf		Crude [96]		
Celastraceae	*Gymnosporia buchananii* Loes.		Crude [102]			
Celastraceae	*Gymnosporia senegalensis* (Lam.) Loes.		Crude [102]			
Celastraceae	*Maytenus buchananii* (Loes.) R. Wilczek	Root, bark	Crude [102]			
Celastraceae	*Maytenus macrocarpa* (Ruiz & Pav.) Briq.					Triterpenes [144]
Celastraceae	*Maytenus senegalensis* (Lam.) Exell	Stem	Crude [102]	Crude [95]		
Celastraceae	*Salacia chinensis* L.	Stem	Crude [58]			
Celastraceae	*Tripterygium wilfordii* Hook. f.	Root	Salaspermic acid [145]			Crude [146,147] Diterpene [146,148] Sesquiterpene pyridine Alkaloids [147]
Chenopodiaceae	*Chenopodium ambrosioides* L.	Leaf				Crude [82]
Chloranthaceae	*Chloranthus japonicas* Siebold	Whole plant	Disesquiterpenoids [149]	Crude [96]		Crude [150]
Cistaceae	Whole plant	Whole plant				Crude [61]
Cistaceae	*Tuberaria lignose* Samp.	Whole plant				Crude [61]
Cleomaceae	*Cleome viscosa* L.	Seed	Nevirapine [151]	Crude [83]		
Clusiaceae	*Allanblackia stuhlmannii* (Engl.) Engl.					Benzophenone [152]
Clusiaceae	*Calophyllum brasiliense* Cambess.	Leaf	Crude [153] Dipyranocoumarins [154] Coumrains [155]			
Clusiaceae	*Calophyllum cerasiferum* Vesque		Coumarins [156]			
Clusiaceae	*Calophyllum cordato-oblongum* Thwaites		Cordatolide [157]			
Clusiaceae	*Calophyllum inophyllum* L.	Bark	Crude [158]	Crude [158]	Crude [158]	Dipyranocoumarins [159] Inophyllum [160]
Clusiaceae	*Calophyllum lanigerum* Miq.		Calanolide [161]			Calanolide [162] Coumarin [163] Pyranocoumarins [164]
Clusiaceae	*Calophyllum rubiginosum* M.R. Hend. & Wyatt-Sm.	Stem bark				Crude [165]
Clusiaceae	*Calophyllum teysmannii* Miq.					Pyranocoumarins [141]
Clusiaceae	*Clusia quadrangular* Bartlett		Crude [153]			
Clusiaceae	*Garcinia buchneri* Engl.	Stem bark		Crude [166]		
Clusiaceae	*Garcinia gummi-gutta* Roxb.	Leaf	Crude [158]	Crude [158]	Crude [158]	
Clusiaceae	*Garcinia hanburyi* Hook. f.	Root				Xanthone [167]
Clusiaceae	*Garcinia indica* Choisy	Leaf	Crude [158]	Crude [158]	Crude [158]	
Clusiaceae	*Garcinia kingaensis* Engl.	Stem bark		Crude [166]		
Clusiaceae	*Garcinia livingstonei* T. Anderson	Fruit				Crude [168]
Clusiaceae	*Garcinia mangostana* L.	Fruit bark	Crude [58]	Crude [169]		
Clusiaceae	*Garcinia semseii* Verdc.	Stem bark		Crude [166]		Crude [168]
Clusiaceae	*Garcinia smeathmanii* (Planch. & Triana) Oliv.	Stem bark		Crude [166]		
Colchicaceae	*Colchicum luteum* Baker	Bulb				Crude [56]
Combretaceae	*Anogeissus acuminata* (Roxb. ex DC.) Guill., Perr. & A. Rich.		Lignans [170]			Crude [170]
Combretaceae	*Combretum adenogonium* Steud. ex A. Rich.	Root, Leaf and Stem bark		Crude [171]		
Combretaceae	*Combretum hartmannianum* C. Schweinf.	Stem	Crude [74]			
Combretaceae	*Combretum molle* R. Br. ex G. Don	Root	Crude [172]			Crude [173]
Combretaceae	*Combretum paniculatum* Vent.	Leaf				Crude [174]
Combretaceae	*Terminalia arjuna* (Roxb. ex DC.) Wight & Arn.	Stem bark		Crude [68,83]		Crude [56]
Combretaceae	*Terminalia bellirica* (Gaertn.) Roxb.	Fruit	Crude [58,175]	Crude [68]		Crude [176]
Combretaceae	*Terminalia chebula* Retz.	Fruit	Crude [58,175]	Crude [68,83]	Galloyl glycosides [177]	Crude [175]
Combretaceae	*Terminalia sericea* Burch. ex DC.		Crude [178]			Crude [179]
Convolvulaceae	*Argyreia nervosa* (Burm. f.) Bojer	Aerial part	Crude [57]			
Convolvulaceae	*Calystegia soldanella* (L.) R. Br.	Leaf, Stem		Crude [96]		
Convolvulaceae	*Cuscuta chinensis* Lam.	Fruit, Stem		Crude [96]		
Convolvulaceae	*Cuscuta japonica* Choisy	Semen		Crude [96]		Crude [66]
Convolvulaceae	*Ipomoea aquatic* Forssk.	Whole plant	Crude [57]			
Convolvulaceae	*Ipomoea cairica* (L.) Sweet	Whole plant	Crude [57]			Lignans [180]
Convolvulaceae	*Ipomoea carnea* Jacq.	Aerial part	Crude [57]			
Convolvulaceae	*Merremia peltata* (L.) Merr.					Crude [181]
Cornaceae	*Cornus walteri* Wangerin	Aerial part		Crude [96]		
Cornaceae	*Camptotheca acuminata* Decne		Rubitecan [182]			
Crassulaceae	*Orostachys japonica* A. Berger	Aerial part		Crude [183]		
Crassulaceae	*Sedum album* L.	Whole plant				Crude [61]
Crassulaceae	*Sedum maximum* Hoffm.	Leaf	Crude [62]			
Crassulaceae	*Sedum polytrichoides* Hemsl.	Whole plant		Crude [96]		
Crassulaceae	*Sedum roseum* Scop.			Crude [96]		
Cucurbitaceae	*Citrullus colocynthis* (L.) Schrad.	Fruit peel	Crude [74]			
Cucurbitaceae	*Gynostemma pentaphyllum* (Thunb.) Makino					Crude [184]
Cucurbitaceae	*Hemsleya endecaphylla* C.Y. Wu	Tuber				Crude [185]
Cucurbitaceae	*Momordica balsamina* L.	Leaf				Crude [186]
Cucurbitaceae	*Momordica charantia* L.	Seed, Fruit				Crude [187]
Cucurbitaceae	*Momordica cochinchinensis* (Lour.) Spreng.	Semen		Crude [96]		Crude [66]
Cucurbitaceae	*Trichosanthes kirilowii* Maxim.	Semen				Crude [66,188]
Cupressaceae	*Cupressus sempervirens* L.					Crude [189]
Cupressaceae	*Platycladus orientalis* (L.) Franco					Crude [66]
Cupressaceae	*Thuja occidentalis* L.					Crude [190]
Cyperaceae	*Bolboschoenus maritimus* (L.) Palla					Crude [66]
Cyperaceae	*Cyperus rotundus* L.	Rhizome		Crude [68]		
Davalliaceae	*Davallia mariesii* T. Moore ex Baker	Root				Crude [66]
Dioscoreaceae	*Dioscorea bulbifera* L.				Flavonoid [191]	
Dioscoreaceae	*Dioscorea hispida* Dennst.	Rhizome		Crude Protease [68]		
Dioscoreaceae	*Dioscorea polystachya* Turcz.					Crude inhibition [66]
Dioscoreaceae	*Dioscorea tokoro* Makino	Root				Crude inhibition [66]
Dipterocarpaceae	*Monotes africana* A. DC.					Crude [192]
Dryopteridaceae	*Cyrtomium fortune* J. Sm.	Rhizome		Crude Protease [68]		
Dryopteridaceae	*Dryopteris crassirhizoma* Nakai	Rhizome	Flavonoid [193]	Triterpenes [194]		
Ebenaceae	*Euclea natalensis* A. DC.		Naphthoquinone [195]			
Ebenaceae	*Diospyros mollis* Griff.	Stem	Crude [58]			
Elaeocarpaceae	*Elaeocarpus grandiflorus* Sm.	Fruit		Crude [68]		
Ephedraceae	*Ephedra americana* Humb. & Bonpl. ex Willd.	Stem				Crude [82]
Ephedraceae	*Ephedra sinica* Stapf	Stem	Crude [196]	Crude [68]		Crude [196]
Equisetaceae	*Equisetum arvense* L.	Stem				Crude [82]
Equisetaceae	*Equisetum giganteum* L.	Stem				Crude [82]
Equisetaceae	*Equisetum hyemale* L.	Aerial part				Crude [66]
Erythroxylaceae	*Erythroxylum citrifolium* A. St.-Hil.	Trunk		Crude [93]		
Eucommiaceae	*Eucommia ulmoides* Oliv.	Stem bark				Crude [66]
Euphorbiaceae	*Acalypha macrostachya* Jacq.	Leaf				Crude [93]
Euphorbiaceae	*Alchornea cordifolia* (Schumach. & Thonn.) Müll. Arg.	Leaf				Crude [80]
Euphorbiaceae	*Baliospermum solanifolium* (Geiseler) Suresh			Crude [99]		
Euphorbiaceae	*Chamaesyce hyssopifolia* (L.) Small	Whole plant	Crude [93]	Crude [93]		
Euphorbiaceae	*Croton billbergianus* Müll. Arg.	Trunk				Crude [93]
Euphorbiaceae	*Croton gratissimus* Burch.		Crude [74]			
Euphorbiaceae	*Croton tiglium* L.	Seed				Crude [197]
Euphorbiaceae	*Croton zambesicus* Müll. Arg.	Seed	Crude [74]	Crude [95]		
Euphorbiaceae	*Euphorbia erythradenia* Boiss.	Aerial part				Triterpene [198]
Euphorbiaceae	*Euphorbia granulate* Forssk.	Leaf		Crude [95]		
Euphorbiaceae	*Euphorbia hirta* L.	Whole plant	Crude [58]			
Euphorbiaceae	*Euphorbia hyssopifolia* L.	Whole plant	Crude [93]	Crude [93]		
Euphorbiaceae	*Euphorbia kansui* T.N. Liou ex S.B. Ho					Crude [199]
Euphorbiaceae	*Euphorbia neriifolia* L.	Stem bark				Diterpenoids [200,201]
Euphorbiaceae	*Euphorbia polyacantha* Boiss.		Crude [74]			
Euphorbiaceae	*Euphorbia prostrate* Aiton			Crude [95]		
Euphorbiaceae	*Euphorbia thi* Schweinf.	Aerial part	Crude [74]			
Euphorbiaceae	*Homalanthus nutans* (G. Forst.) Guill.					Prostratin [202]
Euphorbiaceae	*Jatropha curcas* L.	Leaf	Crude [93]	Crude [93]		Crude [80,93]
Euphorbiaceae	*Mallotus japonicus* (L.f.) Müll.Arg.		Tannins [203]			
Euphorbiaceae	*Mallotus philippensis* (Lam.) Müll. Arg.	Flower	Crude [58]			
Euphorbiaceae	*Maprounea africana* Müll. Arg.	Leaf	Xanthone [204] Triterpene [205]			Crude [80] Triterpene [205]
Euphorbiaceae	*Neoshirakia japonica* (Siebold & Zucc.) Esser	Leaf		Crude [96]		
Euphorbiaceae	*Ricinus communis* L.	Leaf	Lectins [206]	Crude [83]		Crude [207]
Euphorbiaceae	*Sapium indicum* Willd.	Fruit	Crude [58]			
Euphorbiaceae	*Shirakiopsis indica* (Willd.) Esser		Crude [58]			
Euphorbiaceae	*Trigonostemon thyrsoideus* Stapf	Stem				Diterpenoid [208,209]
Fabaceae	*Abrus precatorius* L.	Seed		Saponins [210]		Crude [211]
Fabaceae	*Acacia catechu* (L. f.) Willd.	Resin	Crude [58]			Crude [212]
Fabaceae	*Acacia mellifera* (Vahl) Benth.	Stem bark	Crude [102]			
Fabaceae	*Acacia nilotica* (L.) Willd. ex Delile	Bark		Crude [95]		
Fabaceae	*Albizia gummifera* (J.F. Gmel.) C.A. Sm.	Stem bark	Crude [102]			
Fabaceae	*Albizia procera* (Roxb.) Benth.				Crude [113]	Crude [113]
Fabaceae	*Astragalus propinquus* Schischk.	Aerial part		Crude [68]		Crude [51]
Fabaceae	*Astragalus spinosus* Muschl.	Aerial part				Triterpene [213]
Fabaceae	*Bauhinia strychnifolia* Craib				Crude [113]	
Fabaceae	*Bauhinia variegata* L.		Crude [134]			
Fabaceae	*Butea monosperma* (Lam.) Taub.	Root				Crude [56]
Fabaceae	*Caesalpinia bonduc* (L.) Roxb.	Seed		Crude [83]		
Fabaceae	*Caesalpinia sappan* L.	Stem	Crude [58]		Crude [113]	Crude [66]
Fabaceae	*Canavalia gladiate* (Jacq.) DC.		Crude [134]			
Fabaceae	*Cassia fistula* L.	Bark		Crude [68,83]		
Fabaceae	*Castanospermum austral* A. Cunn. & C. Fraser					Alkaloid [214]
Fabaceae	*Cullen corylifolium* (L.) Medik.					Crude [66]
Fabaceae	*Detarium microcarpum* Guill. & Perr.					Flavonoids [215]
Fabaceae	*Elephantorrhiza elephantine* (Burch.) Skeels	Bulb				Crude [79]
Fabaceae	*Erythrina abyssinica* Lam.	Bark	Crude [74] [102]			Alkaloids [216]
Fabaceae	*Erythrina senegalensis* DC.				Flavonoids [217]	
Fabaceae	*Euchresta formosana* (Hayata) Ohwi					Crude [218]
Fabaceae	*Gleditsia japonica* Miq.	Fruit				Saponin [219]
Fabaceae	*Glycine max* (L.) Merr.		Crude [134]			
Fabaceae	*Glycyrrhiza glabra* L.			Crude [220]		Crude [56,221]
Fabaceae	*Glycyrrhiza uralensis* Fisch. ex DC.					Crude [222]
Fabaceae	*Gymnocladus chinensis* Baill.	Fruit				Saponin [219]
Fabaceae	*Hylodendron gabunense* Taub.					Crude [223]
Fabaceae	*Lespedeza juncea* (L. f.) Pers.	Whole plant		Crude [96]		
Fabaceae	*Lespedeza tomentosa* (Thunb.) Siebold ex Maxim.	Leaf		Crude [96]		
Fabaceae	*Melilotus suaveolens* Ledeb.	Whole plant		Crude [96]		
Fabaceae	*Millettia erythrocalyx* Gagnep.	Leaf				Flavonoid [224]
Fabaceae	*Peltophorum africanum* Sond.	Stem bark	Crude [172]		Crude [172]	Betulinic acid [225]
Fabaceae	*Phaseolus vulgaris* L.	Seed	Lectin [226]			[223]
Fabaceae	*Pongamia pinnata* (L.) Pierre	Bark	Flavonoids [227]	Crude [83]		
Fabaceae	*Prosopis glandulosa* Torr.	Leaf				Oleanolic acid [228]
Fabaceae	*Psoralea glandulosa* L.	Leaf				Crude [82]
Fabaceae	*Pterocarpus marsupium* Roxb.		Crude [229]			
Fabaceae	*Pueraria montana* (Lour.) Merr.			Crude [60]		Crude [66]
Fabaceae	*Saraca indica* L.	Bark		Crude [83]		
Fabaceae	*Securigera securidaca* (L.) Degen & Dorfl.		Kaempferol [230]			
Fabaceae	*Senna alata* Roxb.	Aerial part	Crude [57]			
Fabaceae	*Senna garrettiana* (Craib) H.S.Irwin & Barneby				Crude [113]	
Fabaceae	*Senna obtusifolia* (L.) H.S. Irwin & Barneby	Aerial part		Crude [95]		Crude [231]
Fabaceae	*Senna occidentalis* (L.) Link	Leaf				Crude [56]
Fabaceae	*Sophora flavescens* Aiton	Root	Crude [196]	Crude [60,96]	Crude [105]	Crude [196]
Fabaceae	*Sophora japonica* L.	Flower				Crude [66]
Fabaceae	*Sophora tonkinensis* Gagnep.	Root		Crude [60,68]		
Fabaceae	*Spatholobus suberectus* Dunn	Rhizome		Crude [60,68]	Crude [105]	
Fabaceae	*Styphnolobium japonicum* (L.) Schott	Flower bud		Crude [68]		Crude [66]
Fabaceae	*Sutherlandia frutescens* (L.) R. Br.		Crude [132]			
Fabaceae	*Tephrosia purpurea* (L.) Pers.	Root		Crude [83]		
Fabaceae	*Vigna unguiculata* (L.) Walp.	Seed		Crude [83]		
Fagaceae	*Quercus infectoria* Olivier	Fruit	Crude [58]			
Fagaceae	*Quercus robur* L.		Crude [175]			
Flacourtiaceae	*Hydnocarpus anthelminthicus* Pierre ex Laness.	Semen				Crude [66]
Gentianaceae	*Gentiana asclepiadea* L.	Leaf	Crude [62]			
Gentianaceae	*Gentiana macrophylla* Pall.	Root		Crude [68]		
Gentianaceae	*Gentiana scabra* Bunge	Root		Crude [68]		
Gentianaceae	*Swertia bimaculata* (Siebold & Zucc.) Hook. f. & Thomson ex C.B. Clarke					Sesterterpenoid [232]
Gentianaceae	*Swertia franchetiana* Harry Sm.	Root	Xanthone [204]			Xanthone [233]
Gentianaceae	*Swertia punicea* Hemsl.					Xanthone [234]
Gentianaceae	*Tripterospermum lanceolatum* (Hayata) H. Hara ex Satake		Crude [235]			
Gesneriaceae	*Drymonia serrulata* (Jacq.) Mart.	Leaf				Crude [93]
Ginkgoaceae	*Ginkgo biloba* L.	Semen	Crude [236]	Crude [236] Ginkgolic acid [237]		Crude [66]
Gunneraceae	*Gunnera magellanica* Lam.	Stem				Crude [82]
Hydrangeaceae	*Philadelphus schrenkii* Rupr.	Stem		Crude [96]		
Hydrocharitaceae	*Thalassia testudunum* Banks & Sol. ex K.D. Koenig					Crude [238]
Hypericaceae	*Cratoxylum arborescens* Blume	Leaf				Xanthones [239]
Hypericaceae	*Hypericum capitatum* Choisy					Crude [240]
Hypericaceae	*Hypericum hircinum* L.		Crude [241]			
Hypericaceae	*Hypericum perforatum* L.					Crude [242]
Hypericaceae	*Vismia baccifera* (L.) Triana & Planch.		Crude [155]			
Hypericaceae	*Vismia cayennensis* (Jacq.) Pers.	Leaf				Crude [243]
Hypoxidaceae	*Hypoxis hemerocallidea* Fisch., C.A. Mey. & Avé-Lall.					Crude [244]
Hypoxidaceae	*Hypoxis sobolifera* Jacq.	Corm	Crude [75]	Crude [75]		
Iridaceae	*Aristea ecklonii* Baker					
Iridaceae	*Eleutherine bulbosa* (Mill.) Urb.	Bulb				Naphthoquinone [245]
Iridaceae	*Iris domestica* (L.) Goldblatt & Mabb.			Crude [68]		
Juglandaceae	*Juglans mandshurica* Maxim.	Bark		Crude [96]		Glycosides [246]
Lamiaceae	*Aegiphila anomala* Pittier	Leaf	Crude [93]			
Lamiaceae	*Agastache rugosa* (Fisch. & C.A. Mey.) Kuntze	Whole plant		Crude [60,96]	Crude [247]	Crude [248]
Lamiaceae	*Ajuga decumbens* Thunb.		Crude [249]			
Lamiaceae	*Anisomeles indica* (L.) Kuntze					Diterpenoid [250]
Lamiaceae	*Clinopodium bolivianum* (Benth.) Kuntze	Leaf				Crude [82]
Lamiaceae	*Clinopodium chinense* (Benth.) Kuntze	Whole plant		Crude [96]		
Lamiaceae	*Coleus forskohlii* (Willd.) Briq.	Aerial part				Crude [56,251]
Lamiaceae	*Cornutia grandifolia* (Schltdl. & Cham.) Schauer	Trunk				Crude [93]
Lamiaceae	*Cornutia pyramidata* L.					Crude [93]
Lamiaceae	*Hyptis capitata* Jacq.	Whole plant				Oleanolic acid [228]
Lamiaceae	*Hyptis lantanifolia* Poit.	Aerial part	Crude [93]	Crude [93]		
Lamiaceae	*Hyssopus officinalis* L.	Leaf	Crude [252]			
Lamiaceae	*Isodon excisus* (Maxim.) Kudô	Whole plant		Crude [96]		
Lamiaceae	*Isodon inflexus* (Thunb.) Kudô			Crude [96]		
Lamiaceae	*Leonotis leonurus* (L.) R. Br.	Leaf	Crude [75]	Crude [75]		
Lamiaceae	*Leonurus japonicas* Houtt.	Semen				Crude [66]
Lamiaceae	*Leonurus sibiricus* L.	Aerial part		Crude [96]		
Lamiaceae	*Lycopus lucidus* Turcz. ex Benth.	Whole plant		Crude [68]		
Lamiaceae	*Marrubium vulgare* L.	Leaf				Crude [82]
Lamiaceae	*Meehania urticifolia* (Miq.) Makino	Whole plant		Crude [96]		
Lamiaceae	*Melissa officinalis* L.	Whole plant				Crude [253]
Lamiaceae	*Mentha arvensis* L.	Leaf				Crude [66]
Lamiaceae	*Mentha canadensis* L.	Whole plant		Crude [60,68]		
Lamiaceae	*Mentha longifolia* (L.) Huds.					Crude [254]
Lamiaceae	*Minthostachys mollis* Griseb.	Leaf				Crude [82]
Lamiaceae	*Mosla scabra* (Thunb.) C.Y. Wu & H.W. Li	Whole plant		Crude [96]		
Lamiaceae	*Ocimum basilicum* L.	Leaf	Crude [58]			Crude [255]
Lamiaceae	*Ocimum kilimandscharicum* Baker ex Gürke		Crude [255]			
Lamiaceae	*Ocimum labiatum* (N.E. Br.) A.J. Paton					Triterpenoid [256]
Lamiaceae	*Ocimum tenuiflorum* L.	Leaf	Crude [54,58]			
Lamiaceae	*Perilla frutescens* (L.) Britton	Leaf		Crude [60]		Crude [66]
Lamiaceae	*Plectranthus amboinicus* (Lour.) Spreng.	Leaf	Crude [229]	Crude [83,99]		
Lamiaceae	*Plectranthus barbatus* Andrews					Crude [257]
Lamiaceae	*Pogostemon heyneanus* Benth.	Leaf		Crude [83]		
Lamiaceae	*Prunella vulgaris* L.	Whole plant		Crude [60]	Crude [105]	Crude [51,72,258]
Lamiaceae	*Rosmarinus officinalis* L.			Crude [259]		
Lamiaceae	*Salvia haenkei* Benth.					Crude [82]
Lamiaceae	*Salvia miltiorrhiza* Bunge	Root	Crude [260]	Crude Protease [68]	Crude [261]	
Lamiaceae	*Salvia officinalis* L.	Leaf	Crude [262]		Coumarin [263]	Crude [264]
Lamiaceae	*Salvia punctate* Ruiz & Pav.					Crude [82]
Lamiaceae	*Salvia revolute* Ruiz & Pav.					Crude [82]
Lamiaceae	*Salvia yunnanensis* C.H. Wright	Root				Polyphenol [265]
Lamiaceae	*Satureja cuneifolia* Ten.	Whole plant				Crude [61]
Lamiaceae	*Satureja obovate* Lag.	Whole plant				Crude [61]
Lamiaceae	*Scutellaria baicalensis* Georgi	Root		Crude [60,68]		Flavonoid [266]
Lamiaceae	*Teucrium buxifolium* Schreb.	Whole plant				Crude [61]
Lamiaceae	*Vitex glabrata* R. Br.	Branche	Crude [57]			
Lamiaceae	*Vitex negundo* L.	Aerial part	Crude [57]			
Lamiaceae	*Vitex trifolia* L.	Aerial part	Crude [57]			Crude [66]
Lardizabalaceae	*Akebia quinata* (Houtt.) Decne.	Lignum				Crude [66]
Lardizabalaceae	*Stauntonia obovatifoliola* Hayata			Triterpenoid [267]		
Lauraceae	*Cinnamomum loureiroi* Nees	Stem bark	Crude [58]			
Lauraceae	*Cinnamomum verum* J. Presl	Leaf		Crude [83]		
Lauraceae	*Lindera aggregate* (Sims) Kosterm.	Stem		Crude [60]	Crude [268]	Crude [66]
Lauraceae	*Lindera chunii* Merr.				Sesquiterpenoid [269]	
Lauraceae	*Lindera erythrocarpa* Makino	Leaf		Crude [270]		
Lauraceae	*Lindera obtusiloba* Blume	Leaf, Stem		Crude [96]		
Lauraceae	*Litsea glutinosa* (Lour.) C.B. Rob.	Bark		Crude [83]		
Lauraceae	*Litsea verticillata* Hance	Leaf	Crude [58]			Crude [271]
Liliaceae	*Amana edulis* (Miq.) Honda		Crude [196]	Crude [96]		Crude [196]
Liliaceae	*Fritillaria cirrhosa* D. Don	Rhizome		Crude [60]	Crude [105]	
Liliaceae	*Fritillaria thunbergii* Miq.	Rhizome		Crude [68]		
Loasaceae	*Caiophora pentlandii* (Paxton ex Graham) G. Don ex Loudon	Leaf				Crude [82]
Loganiaceae	*Strychnos ignatii* P.J. Bergius	Semen				Crude [66]
Loganiaceae	*Strychnos nuxvomica* L.	Seed	Crude [58]			
Loganiaceae	*Strychnos potatorum* L. f.	Seed		Crude [83]		
Loranthaceae	*Scurrula parasitica* L.	Aerial part		Crude [68]		
Lycopodiaceae	*Lycopodium japonicum* Thunb.					Alkaloids [272]
Lythraceae	*Lawsonia inermis* L.	Aerial part	Crude [58]			
Lythraceae	*Lythrum salicaria* L.	Leaf	Crude [62]			
Lythraceae	*Punica granatum* L.	Fruit bark	Crude [58]	Crude [68,83]		
Lythraceae	*Woodfordia fruticosa* (L.) Kurz	Flower		Crude [68]		
Magnoliaceae	*Magnolia biondii* Pamp.	Flower bud		Crude [68]		
Magnoliaceae	*Magnolia denudate* Desr.	Flower		Crude [96]		
Magnoliaceae	*Magnolia obovate* Thunb.	Bark		Crude [68]		
Magnoliaceae	*Magnolia officinalis* Rehder & E.H. Wilson	Bark		Crude [68]		
Malpighiaceae	*Tetrapterys goudotiana* Triana & Planch.		Crude [93]	Crude [93]		
Malvaceae	*Adansonia digitata* L.	Leaf	Crude [273]	Crude [273]		
Malvaceae	*Corchoropsis tomentosa* (Thunb.) Makino	Aerial part		Crude [96]		
Malvaceae	*Grewia mollis* Juss.	Root	Crude [102]			
Malvaceae	*Hibiscus sabdariffa* L.	Flower	Crude [58]			
Malvaceae	*Pavonia schiedeana* Steud.	Aerial part	Crude [93]			
Malvaceae	*Sida cordata* (Burm. f.) Borss. Waalk.	Root		Crude [83]		Polyphenols [274]
Malvaceae	*Sida mysorensis* Wight & Arn.	Seed		Crude [68]		Polyphenols [274]
Malvaceae	*Sida rhombifolia* L.	Leaf				Crude [80] Polyphenols [274]
Malvaceae	*Thespesia populnea* (L.) Sol. ex Corrêa					Crude [275]
Malvaceae	*Tilia amurensis* Rupr.	Leaf, Stem		Crude [96]		
Malvaceae	*Waltheria indica*	Branch		Crude [93]		
Meliaceae	*Aglaia lawii* (Wight) C.J. Saldanha	Leaf			Crude [276]	
Meliaceae	*Azadirachta indica* A. Juss.	Leaf	Crude [58,102]	Crude [83,95]		
Meliaceae	*Khaya senegalensis* (Desr.) A. Juss.			Crude [95]		
Meliaceae	*Melia azedarach* L.	Fruit	Crude [102]			Crude [66]
Meliaceae	*Swietenia macrophylla* King					Crude [277]
Meliaceae	*Swietenia mahagoni* (L.) Jacq.	Bark		Crude [278]		
Meliaceae	*Trichilia emetic* Vahl			Crude [95]		
Melianthaceae	*Bersama abyssinica* Fresen.	Root			Crude [174]	
Menispermaceae	*Coscinium fenestratum* Colebr.	Gall	Crude [158]	Crude [83,158]	Crude [158]	
Menispermaceae	*Pericampylus glaucus* (Lam.) Merr.	Aerial part				Alkaloids [279]
Menispermaceae	*Sinomenium acutum* (Thunb.) Rehder & E.H. Wilson	Root		Crude [96]		
Menispermaceae	*Stephania cephalantha* Hayata	Root				Crude [280]
Menispermaceae	*Tinospora crispa* (L.) Hook. f. & Thomson	Vine	Crude [57]		Crude [281]	
Menispermaceae	*Tinospora sinensis* (Lour.) Merr.	Stem bark	Crude [54]			Crude [56]
Menyanthaceae	*Nymphoides peltata* (S.G. Gmel.) Kuntze	Whole plant				Crude [66]
Monimiaceae	*Boldea fragrans* Endl.					Crude [82]
Moraceae	*Artocarpus heterophyllus* Lam.	Seed	Crude [58]			
Moraceae	*Ficus carica* L.	Leaf				Crude [124]
Moraceae	*Ficus edelfeltii* King	Bark		Crude [68]		
Moraceae	*Ficus racemosa* L.	Bark			Crude [282]	
Moraceae	*Ficus religiosa* L.	Bark		Crude [83]		
Moraceae	*Maclura cochinchinensis* (Lour.) Corner	Stem	Crude [58]			
Moraceae	*Maclura tinctoria* (L.) D. Don ex Steud.					Xanthones [283]
Moraceae	*Morus alba* L.	Stem bark				Crude [66]
Moringaceae	*Moringa oleifera* Lam.	Seed	Crude [58,74]			
Musaceae	*Musa acuminata* Colla	Fruit				Lectin [284]
Myricaceae	*Morella salicifolia* (Hochst. ex A. Rich.) Verdc. & Polhill	Root bark	Crude [102]			
Myricaceae	*Myrica salicifolia* Hochst. ex A. Rich.	Root bark	Crude [102]			
Myristicaceae	*Myristica fragrans* Houtt.	Stem	Crude [58]	Crude [83]		
Myrothamnaceae	*Myrothamnus flabellifolius* Welw.	Leaf	Polyphenol [285]			
Myrtaceae	*Corymbia citriodora* (Hook.) K.D. Hill & L.A.S. Johnson					Crude [80]
Myrtaceae	*Eucalyptus citriodora* Hook.	Leaf				Crude [80]
Myrtaceae	*Eugenia hiemalis* Cambess.					Glycosides [286]
Myrtaceae	*Psidium guajava* L.					Saponin [287]
Myrtaceae	*Syzygium aromaticum* (L.) Merr. & L.M. Perry					Crude [288]
Myrtaceae	*Syzygium claviflorum* (Roxb.) Wall. ex A.M. Cowan & Cowan	Leaf				Oleanolic acid [228]
Myrtaceae	*Syzygium cumini* (L.) Skeels	Bark		Crude [83]		
Nelumbonaceae	*Nelumbo nucifera* Gaertn.	Leaf				Crude [289]
Nyctaginaceae	*Boerhavia caribaea* Jacq.	Root				Crude [82]
Nyctaginaceae	*Boerhavia diffusa* L.					Crude [290]
Nyctaginaceae	*Boerhavia erecta* L.				Glycosides [291]	
Ochnaceae	*Ochna integerrima* (Lour.) Merr.	Leaf				Flavonoids [292]
Olacaceae	*Heisteria spruceana* Engl.	Bark				Crude [82]
Olacaceae	*Ximenia americana* L.	Stem bark				Crude [174]
Olacaceae	*Ximenia caffra* Sond.		Crude [293]			
Oleaceae	*Chionanthus retusus* Lindl. & Paxton			Crude [96]		
Oleaceae	*Ligustrum lucidum* W.T. Aiton	Fruit		Crude [60]	Crude [105]	
Onagraceae	*Epilobium angustifolium* L.	Leaf	Crude [62]			
Onagraceae	*Oenothera erythrosepala* (Borbás) Borbás	Leaf				Oenothein [294]
Onocleaceae	*Matteuccia struthiopteris* (L.) Tod.	Rhizome		Crude [68]		
Orchidaceae	*Arundina graminifolia* (D. Don) Hochr.	Whole plant				Crude [295]
Orchidaceae	*Bletilla striata* (Thunb.) Rchb. f.	Root				Crude [66]
Orchidaceae	*Dendrobium moniliforme* (L.) Sw.	Whole plant				Crude [66]
Orobanchaceae	*Melampyrum roseum* Maxim.	Whole plant		Crude [96]		
Orobanchaceae	*Pedicularis resupinata* L.	Whole plant		Crude [96]		
Orobanchaceae	*Rehmannia glutinosa* (Gaertn.) Libosch. ex Fisch. & C.A. Mey.	Root				Crude [66]
Paeoniaceae	*Paeonia lactiflora* Pall.					Crude [66]
Paeoniaceae	*Paeonia suffruticosa* Andrews	Root		Crude [60,68]	Crude [105]	
Papaveraceae	*Argemone mexicana* L.	Leaf				Crude [56]
Papaveraceae	*Papaver somniferum* L.	Seed				Crude [56]
Parmeliaceae	*Usnea florida* (L.) Weber ex F.H. Wigg.	Whole plant				Crude [82]
Pentaphylacaceae	*Ternstroemia gymnanthera* (Wight & Arn.) Sprague	Aerial part				Oleanolic acid [228]
Phrymaceae	*Phryma leptostachya* L.	Whole plant		Crude [96]		
Phyllanthaceae	*Aporosa cardiosperma* (Gaertn.) Merr.			Crude [99]		
Phyllanthaceae	*Bridelia ferruginea* Benth.	Stem bark				Crude [80]
Phyllanthaceae	*Bridelia micrantha* (Hochst.) Baill.	Root	Crude [296]			
Phyllanthaceae	*Hymenocardia acida* Tul.	Leaf				Crude [80]
Phyllanthaceae	*Phyllanthus amarus* Schumach. & Thonn.		Crude [297]			
Phyllanthaceae	*Phyllanthus emblica* L.	Fruit	Crude [83]			Crude [175]
Phyllanthaceae	*Phyllanthus myrtifolius* Moon ex Hook. f.		Lignans [137]			
Phyllanthaceae	*Phyllanthus niruri* L.		Crude [298]			
Phyllanthaceae	*Phyllanthus sellowianus* (Klotzsch) Müll. Arg.		Crude [122]			Crude [108]
Pinaceae	*Pinus nigra* J.F. Arnold	Seed				Crude [299]
Pinaceae	*Pinus parviflora* Siebold & Zucc.	Cone				Crude [300]
Piperaceae	*Piper aduncum* L.					Crude [82]
Piperaceae	*Piper elongatum* Vahl	Leaf				Crude [82]
Piperaceae	*Piper longum* L.	Fruit				Crude c
Plantaginaceae	*Digitalis purpurea* L.	Leaf				Crude [82]
Plantaginaceae	*Scoparia dulcis* L.	Leaf	Crude [301]			
Plumbaginaceae	*Plumbago indica* L.	Root	Crude [58]			
Poaceae	*Chrysopogon zizanioides* (L.) Roberty	Root		Crude [83]		
Poaceae	*Coix lacryma* L.	Seed		Crude [68]		
Poaceae	*Cortaderia rudiuscula* Stapf	Leaf				Crude [82]
Poaceae	*Saccharum officinarum* L.	Stem	Crude [58]			
Poaceae	*Sasa borealis* (Hack.) Makino & Shibata	Whole plant		Crude [96]		
Polemoniaceae	*Cantua hibrida* Herrera	Leaf				Crude [82]
Polygalaceae	*Polygala tenuifolia* Willd.	Root				Crude [66]
Polygonaceae	*Muehlenbeckia fruticulosa* (Walp.) Standl.	Leaf				Crude [82]
Polygonaceae	*Persicaria tinctoria* (Aiton) H. Gross	Whole plant		Crude [96]		
Polygonaceae	*Polygonum aviculare* L.	Aerial part				Crude [66]
Polygonaceae	*Polygonum senticosum* (Meisn.) Franch. & Sav.	Whole plant		Crude [96]		
Polygonaceae	*Reynoutria japonica* Houtt.	Root		Crude [68]		
Polygonaceae	*Reynoutria multiflora* (Thunb.) Moldenke			Crude [60]	Crude [105]	
Polygonaceae	*Rheum palmatum* L.	Rhizome	Sennoside [302]	Crude [68] Sennoside [302]	Sennoside [302]	
Polygonaceae	*Rheum tanguticum* Maxim. ex Balf.					Glycosides [303]
Polygonaceae	*Rumex crispus* L.	Root				Crude [82]
Polygonaceae	*Rumex cyprius* Murb.		Crude [175]			
Polygonaceae	*Rumex frutescens* Thouars	Root				Crude [82]
Polygonaceae	*Rumex nepalensis* Spreng.					Crude [111]
Polygonaceae	*Rumex peruanus* Rech. f.	Leaf				Crude [82]
Polypodiaceae	*Drynaria roosii* Nakaike	Rhisome		Crude [68]		
Polypodiaceae	*Pleopeltis pycnocarpa* (C. Chr.) A.R. Sm.					Crude [82]
Polypodiaceae	*Polypodium pycnocarpum* C. Chr.	Root				Crude [82]
Polypodiaceae	*Pyrrosia lingua* (Thunb.) Farw.	Aerial part				Crude [66]
Polypodiaceae	*Polytrichum commune* Hedw.		Crude [62]			
Portulacaceae	*Portulaca oleracea* L.	Aerial part		Crude [68]		
Primulaceae	*Ardisia japonica* (Thunb.) Blume	Aerial part				Crude [304]
Primulaceae	*Embelia ribes* Burm. f.	Fruit				Crude [56]
Proteaceae	*Conospermum incurvum* Lindl.					Crude [305]
Ranunculaceae	*Aconitum ferox* Wall. ex Ser.	Tuber		Crude [83]		
Ranunculaceae	*Aconitum jaluense* Kom.	Root				Crude [66]
Ranunculaceae	*Aconitum uchiyamai* Nakai	Root		Crude [96]		
Ranunculaceae	*Actaea heracleifolia* (Kom.) J. Compton	Rhizome		Crude [68]		
Ranunculaceae	*Anemone chinensis* Bunge	Root		Crude [68]		
Ranunculaceae	*Clematis chinensis* Osbeck	Root		Crude [60,68]		
Ranunculaceae	*Clematis mandschurica* Max.			Crude [96]		
Ranunculaceae	*Coptis chinensis* Franch.	Rhizome		Crude [60,68]	Crude [105]	Crude [72]
Ranunculaceae	*Nigella sativa* L.	Seed		Crude [83]		
Ranunculaceae	*Pulsatilla cernua* (Thunb.) Bercht. ex J. Presl	Root				Crude [66]
Resedaceae	*Reseda lutea* L.	Whole plant				Crude [61]
Resedaceae	*Reseda suffruticosa* Loefl.	Whole plant				Crude [61]
Rhamnaceae	*Berchemia berchemiifolia* (Makino) Koidz.	Bark		Crude [96,270]		
Rhamnaceae	*Rhamnus staddo* A. Rich.		Crude [102]			
Rhamnaceae	*Ziziphus spina-christi* (L.) Desf.	Fruit	Crude [74]			
Rhizophoraceae	*Rhizophora mucronata* Lam.	Leaf	Crude [54]	Crude [55]		
Rosaceae	*Agrimonia pilosa* Ledeb.	Whole plant		Crude [96]		
Rosaceae	*Alchemilla andina* (L.M. Perry) J.F. Macbr.	Stem				Crude [82]
Rosaceae	*Chaenomeles sinensis* (Thouin) Koehne	Fruit				Crude [66]
Rosaceae	*Crataegus pinnatifida* Bunge	Leaf		Crude [96] Triterpenes [306]		
Rosaceae	*Eriobotrya japonica* (Thunb.) Lindl.	Leaf		Crude [96]		Crude [66]
Rosaceae	*Geum macrophyllum* Willd.	Whole plant		Crude [68]		
Rosaceae	*Malus baccata* (L.) Borkh.	Stem		Crude [96]		
Rosaceae	*Malus sieboldii* (Regel) Rehder	Stem		Crude [96]		
Rosaceae	*Prunus africana* (Hook. f.) Kalkman	Stem bark	Crude [102]			
Rosaceae	*Prunus armeniaca* L.	Seed		Crude [68]		
Rosaceae	*Prunus mume* (Siebold) Siebold & Zucc.	Fruit		Crude [68]		
Rosaceae	*Prunus persica* (L.) Batsch	Semen				Crude [66]
Rosaceae	*Prunus yedoensis* Matsum.	Stem bark				Crude [66]
Rosaceae	*Rosa damascena* Mill.					Crude [307]
Rosaceae	*Rosa davurica* Pall.			Crude [308]		
Rosaceae	*Rosa laevigata* Michx.	Fruit				Crude [66]
Rosaceae	*Rosa woodsii* Lindl.	Leaf				Oleanolic acid [228]
Rosaceae	*Sanguisorba minor* Scop.	Whole plant				Crude [61]
Rosaceae	*Sanguisorba officinalis* L.	Root		Crude [96]		Crude [309]
Rosaceae	*Sorbus commixta* Hedl.	Stem		Crude [96]		
Rosaceae	*Stephanandra incise* (Thunb.) Siebold & Zucc. ex Zabel			Crude [96]		
Rubiaceae	*Canthium coromandelicum* (Burm.f.) Alston	Leaf	Crude [310]			
Rubiaceae	*Cinchona pubescens* Vahl	Bark				Crude [82]
Rubiaceae	*Cruciata glabra* Ehrend.		Crude [62]			
Rubiaceae	*Galium aparine* L.	Leaf	Crude [62]			
Rubiaceae	*Galium mollugo* L.	Leaf	Crude [62]			
Rubiaceae	*Galium verum* L.	Whole plant		Crude [96]		
Rubiaceae	*Gardenia ternifolia* Schumach. & Thonn.		Crude [74]			
Rubiaceae	*Gardenia tubifera* Wall. ex Roxb.	Leaf	Cycloartanes [311]			
Rubiaceae	*Hedyotis corymbosa* (L.) Lam.			Crude [99]		
Rubiaceae	*Hedyotis diffusa* Willd.	Aerial part				Crude [66]
Rubiaceae	*Morinda citrifolia* L.	Leaf	Crude [158]	Crude [158]	Crude [158]	
Rubiaceae	*Oldenlandia diffusa* (Willd.) Roxb.	Whole plant		Crude [60,68]	Crude [105]	
Rubiaceae	*Oldenlandia herbacea* (L.) Roxb.	Root		Crude [83]		
Rubiaceae	*Rubia cordifolia* L.	Root	Crude [229]			Crude [56]
Rubiaceae	*Sarcocephalus latifolius* (Sm.) Bruce			Crude [95]		Crude [312]
Rutaceae	*Aegle marmelos* (L.) Corrêa	Leaf		Crude [83]		Crude [56]
Rutaceae	*Citrus hystrix* DC.	Fruit bark	Crude [58]			
Rutaceae	*Clausena anisata*	root	Crude [102]			Crude [313]
Rutaceae	*Clausena excavate* (Willd.) Hook. f. ex Benth.	Aerial part	Crude [57]			Limonoid [314]
Rutaceae	*Dictamnus albus* L.	Root bark		Crude [68]		
Rutaceae	*Murraya koenigii* (L.) Spreng.	Aerial part	Crude [57]			
Rutaceae	*Phellodendron amurense* Rupr.	Bark		Crude [68]		
Rutaceae	*Tetradium ruticarpum* (A. Juss.) T.G. Hartley			Crude [68]		
Rutaceae	*Toddalia asiatica* (L.) Lam.	Root	Crude [102]			Alkaloid [315]
Rutaceae	*Vepris simplicifolia* (Engl.) Mziray		Crude [102]			
Rutaceae	*Zanthoxylum bungeanum* Maxim.	Fruit peel		Crude [68]		Crude [66]
Rutaceae	*Zanthoxylum chalybeum* Engl.	Root bark	Crude [102]			Crude [211]
Rutaceae	*Zanthoxylum schinifolium* Siebold & Zucc.	Fruit peel		Crude [68,96]		
Salvadoraceae	*Salvadora persica* L.	Stem	Crude [74]	Crude [95]		
Santalaceae	*Phoradendron juniperinum* Engelm. ex A. Gray	Whole plant				Oleanolic acid [228]
Santalaceae	*Viscum album* L.	Flower				Crude [118]
Sapindaceae	*Acer okamotoanum* Nakai	Leaf			Flavonoid [316]	
Sapindaceae	*Acer pictum* Thunb.	Stem		Crude [96]		
Sapindaceae	*Aesculus chinensis* Bunge	Seed				Triterpenoid [317]
Sapindaceae	*Aesculus turbinate* Blume	Fruit		Crude [96]		
Sapindaceae	*Allophylus cobbe* (L.) Raeusch.	Leaf			Crude [318]	
Sapindaceae	*Dodonaea viscosa* Jacq.	Leaf				Crude [82,174]
Sapindaceae	*Koelreuteria paniculata* Laxm.	Stem		Crude [96]		
Sapindaceae	*Nephelium lappaceum* L.	Seed	Crude [319]			
Sapindaceae	*Serjania mexicana* (L.) Willd.	Whole plant		Crude [93]		
Sapotaceae	*Madhuca longifolia* (J. Koenig ex L.) J.F. Macbr.	Bark				Crude [56]
Sapotaceae	*Mimusops elengi* L.	Bark	Crude [320]	Crude [83]		Saponin [321]
Sapotaceae	*Tieghemella heckelii* Pierre ex A. Chev.	Leaf				Crude [318]
Sauruaceae	*Houttuynia cordata* Thunb.	Aerial part				Crude [66,322]
Sauruaceae	*Saururus chinensis* (Lour.) Baill.	Rhizome				Lignans [323]
Saxifragaceae	*Astilbe grandis* Stapf ex E.H. Wilson	Aerial part		Crude [96]		
Saxifragaceae	*Astilbe rubra* Hook. f. & Thomson ex Hook.	Whole plant		Crude [96]		
Schisandraceae	*Illicium verum* Hook. f.	Root				Phytochemicals [324]
Schisandraceae	*Kadsura angustifolia* A.C. Sm.					Lignans [325]
Schisandraceae	*Kadsura heteroclite* (Roxb.) Craib			Triterpenoid [326]		Crude [327]
Schisandraceae	*Kadsura longipedunculata* Finet & Gagnep.			Lignans [328]		
Schisandraceae	*Schisandra chinensis* (Turcz.) Baill.	Fruit		Protease [68]		
Schisandraceae	*Schisandra lancifolia* (Rehder & E.H. Wilson) A.C. Sm.	Leaf, Stem				Triterpenoid [329] Nortriterpenoid [330]
Schisandraceae	*Schisandra propinqua* Hook. f. & Thomson	Aerial part				Lignans [331]
Schisandraceae	*Schisandra rubriflora* (Franch.) Rehder & E.H. Wilson					Lignans [332]
Schisandraceae	*Schisandra sphaerandra* Stapf	Stem	Triterpenoid [333]			Triterpenoid [333]
Schisandraceae	*Schisandra sphenanthera* Rehder & E.H. Wilson	Leaf, Stem				Nortriterpenoid [334] Triterpenoids [335]
Schisandraceae	*Schisandra wilsoniana* A.C. Sm.	Fruit				Lignans [336]
Scrophulariaceae	*Buddleja officinalis* Maxim.	Flower				Crude [66]
Scrophulariaceae	*Scrophularia buergeriana* Miq.	Root		Crude [96]		
Scrophulariaceae	*Scrophularia kakudensis* Franch.	Aerial part		Crude [96]		
Scrophulariaceae	*Verbascum densiflorum* Bertol.		Crude [62]			
Scrophulariaceae	*Verbascum thapsiforme* Schrad.		Crude [62]			
Selaginellaceae	*Selaginella tamariscina* (P. Beauv.) Spring	Aerial part				Crude [66]
Simaroubaceae	*Ailanthus altissima* (Mill.) Swingle	Stem bark				Crude [66]
Simaroubaceae	*Brucea javanica* (L.) Merr.	Seed	Crude [58]	Crude [68]		
Simaroubaceae	*Leitneria floridana* Chapm.					Crude [337]
Simaroubaceae	*Quassia amara* L.	Bark				Crude [82]
Smilacacea	*Smilax campestris* Griseb.	Root				Crude [82]
Smilacacea	*Smilax china* L.	Fruit		Crude [96]		Crude [338]
Solanaceae	*Cestrum parqui* L’Hér.	Leaf				Crude [82]
Solanaceae	*Lycium chinense* Mill.	Fruit				Crude [66]
Solanaceae	*Physaliastrum japonicum* (Franch. & Sav.) Honda	Aerial part		Crude [96]		
Solanaceae	*Solanum incanum* L.					Betulinic acid [339]
Solanaceae	*Solanum tomentosum* L.		Crude [340]			
Solanaceae	*Solanum virginianum* L.					Crude [341]
Solanaceae	*Withania somnifera* (L.) Dunal	Root	Crude [54]	Crude [342]		
Staphyleaceae	*Staphylea bumalda* DC.	Whole plant		Crude [96]		
Styracaceae	*Styrax japonicas* Siebold & Zucc.	Stem				Lignins [343]
Styracaceae	*Styrax obassis* Siebold & Zucc.	Stem		Crude [96]		
Tamaricaceae	*Tamarix senegalensis* DC.		Crude [74]			
Taxaceae	*Taxus caespitosa* Nakai	Stem		Crude [96]		
Taxaceae	*Taxus cuspidate* Siebold & Zucc.			Crude [96]		
Theaceae	*Camellia japonica* L.	Leaf		Crude [344]		
Theaceae	*Stewartia koreana* Nakai ex Rehder	Leaf		Crude [96]		
Thymelaeaceae	*Daphne acutiloba* Rehder					Diterpene [345]
Thymelaeaceae	*Daphne feddei* H.Lév.	Leaf, Stem				Lignans [346]
Thymelaeaceae	*Wikstroemia indica* (L.) C.A. Mey.					Crude [347]
Typhaceae	*Typha domingensis* Pers.		Crude [102]			
Ulmaceae	*Ulmus davidiana* Planch.	Leaf, Stem		Crude [96]		
Ulmaceae	*Ulmus pumila* L.	Bark				Crude [66]
Urticaceae	*Myrianthus holstii* Engl.					Lectin [348]
Urticaceae	*Phenax angustifolius* (Kunth) Wedd.	Leaf				Lignans [349]
Urticaceae	*Urtica dioica* L.	Rhizome	Crude [62]			Crude [350]
Urticaceae	*Urtica magellanica* Juss. ex Poir.	Leaf				Crude [82]
Urticaceae	*Urtica urens* L.	Leaf				Crude [82]
Verbenaceae	*Lampaya medicinalis* Phil.	Leaf				Crude [82]
Verbenaceae	*Lippia javanica* (Burm f.) Spreng.		Phytochemicals [351]			
Verbenaceae	*Stachytarpheta jamaicensis* (L.) Vahl	Whole plant	Crude [57]			
Violaceae	*Viola yedoensis* Makino	Whole plant		Crude [60]	Crude [105]	Crude [72]
Vitaceae	*Cissus quadrangularis* L.	Stem	Crude [74]			
Vitaceae	*Vitis vinifera* L.				Phytochemicals [352]	
Xanthorrhoeaceae	*Aloe ferox* Mill.					Crude [353]
Xanthorrhoeaceae	*Aloe vera* (L.) Burm. f.					Crude [354]
Xanthorrhoeaceae	*Asphodelus ramosus* L.	Whole plant				Crude [61]
Xanthorrhoeaceae	*Bulbine alooides* Willd.	Roots	Crude [75]	Crude [75]		
Zingiberaceae	*Alpinia galangal* (L.) Willd.			Crude [355]		Crude [356]
Zingiberaceae	*Alpinia officinarum* Hance	Root		Crude [68]		Crude [66]
Zingiberaceae	*Boesenbergia rotunda* (L.) Mansf.			Phytochemicals [357]		Flavonoid [358]
Zingiberaceae	*Curcuma longa* L.	Rhizome	Crude [58]	Crude [83]	Crude [359]	Crude [66]
Zingiberaceae	*Curcuma zanthorrhiza* Roxb.		Crude [58]			
Zingiberaceae	*Elettaria cardamomum* (L.) Maton	Fruit		Crude [83]		
Zingiberaceae	*Kaempferia parviflora* Wall. ex Baker			Crude [355]		
Zygophyllaceae	*Balanites aegyptiacus* (L.) Delile	Bark		Crude [95]		
Zygophyllaceae	*Larrea tridentata* (Sessé & Moc. ex DC.) Coville					Lignan [360]
Zygophyllaceae	*Tribulus terrestris* L.	Fruit		Crude [95]		Crude [66]

**Table 2:** Plant names which are having synonyms found in theplantlist.org.

Reported Name	Accepted Name
*Aglaia andamanica* Hiern	*Aglaia lawii* (Wight) C.J. Saldanha
*Andropogon muricatus* Retz.	*Chrysopogon zizanioides* (L.) Roberty
*Angelica koreana* Maxim.	*Angelica grosseserrata* Maxim.
*Aporosa lindleyana* (Wight) Baill.	*Aporosa cardiosperma* (Gaertn.) Merr.
*Aster koraiensis* Nakai	*Miyamayomena koraiensis* (Nakai) Kitam.
*Aster scaber* Elliott	*Symphyotrichum undulatum* (L.) G.L. Nesom
*Astilbe chinensis* (Maxim.) Franch. & Sav.	*Astilbe rubra* Hook. f. & Thomson ex Hook.
*Astilbe koreana* (Kom.) Nakai	*Astilbe grandis* Stapf ex E.H. Wilson
*Astragalus membranaceus* Moench	*Astragalus propinquus* Schischk.
*Baliospermum montanum* (Willd.) Müll. Arg.	*Baliospermum solanifolium* (Geiseler) Suresh
*Baphicacanthus cusia* (Nees) Bremek.	*Strobilanthes cusia* (Nees) Kuntze
*Belamcanda chinensis* (L.) Redouté	*Saposhnikovia divaricate* (Turcz.) Schischk.
*Boesenbergia pandurata* (Roxb.) Schltr.	*Boesenbergia rotunda* (L.) Mansf.
*Brassica alboglabra* L.H. Bailey	*Brassica oleracea* L.
*Brassica campestris* L.	*Brassica rapa* L.
*Caesalpinia bonducella* (L.) Fleming	*Caesalpinia bonduc* (L.) Roxb.
*Carissa edulis* (Forssk.) Vahl	*Carissa spinarum* L.
*Cassia garrettiana* Craib	*Senna garrettiana* (Craib) H.S. Irwin & Barneby
*Cassia occidentalis* L.	*Senna occidentalis* (L.) Link
*Chamaesyce hyssopifolia* (L.) Small	*Euphorbia hyssopifolia* L.
*Cimicifuga heracleifolia* Kom.	*Actaea heracleifolia* (Kom.) J. Compton
*Clerodendrum inerme* (L.) Gaertn.	*Volkameria inermis* L.
*Coleus amboinicus* Lour.	*Plectranthus amboinicus* (Lour.) Spreng.
*Curcuma domestica* Valeton	*Curcuma longa* L.
*Cydonia vulgaris* Pers.	*Chaenomeles sinensis* (Thouin) Koehne
*Dictamnus dasycarpus* Turcz.	*Dictamnus albus* L.
*Dodonaea angustifolia* L. f.	*Dodonaea viscosa* Jacq.
*Dolichos biflorus* L.	*Vigna unguiculata* (L.) Walp.
*Drymaria diandra* Blume	*Drymaria cordata* (L.) Willd. ex Schult.
*Drynaria fortunei* (Kunze ex Mett.) J. Sm.	*Drynaria roosii* Nakaike
*Elaeodendron croceum* (Thunb.) DC.	*Cassine crocea* (Thunb.) C. Presl
*Eleutherine americana* (Aubl.) Merr. ex K. Heyne	*Eleutherine bulbosa* (Mill.) Urb.
*Enantia chlorantha* Oliv.	*Annickia chlorantha* (Oliv.) Setten & Maas
*Epinetrum villosum* Troupin	*Albertisia villosa* Forman
*Erythroxylum lucidum* Kunth	*Erythroxylum macrophyllum* Cav.
*Eugenia caryophyllata* Thunb.	*Syzygium aromaticum* (L.) Merr. & L.M. Perry
*Eugenia jambolana* Lam.	*Syzygium cumini* (L.) Skeels
*Eupatorium buniifolium* Hook. ex Arn.	*Acanthostyles buniifolius* (Hook. ex Arn.) R.M. King & H. Rob.
*Euodia ruticarpa* (A. Juss.) Benth.	*Tetradium ruticarpum* (A. Juss.) T.G. Hartley
*Ferula sumbul* (Kauffm.) Hook. f.	*Ferula moschata* (H. Reinsch) Koso-Pol.
*Garcinia cambogia* Roxb.	*Garcinia gummi-gutta* Roxb.
*Garcinia edulis* Exell	*Garcinia buchneri* Engl.
*Garcinia polyantha* Oliv.	*Garcinia smeathmannii* (Planch. & Triana) Oliv.
*Geum japonicum* Thunb.	*Geum macrophyllum* Willd.
*Ginkgo biloba* L.	*Salisburia ginkgo* (L.) Rich.
*Glycosmis montana* Pierre	*Glycosmis lanceolata* (Blume) Teijsm. & Binn. ex Kurz
*Kadsura interior* A.C. Sm.	*Kadsura heteroclite* (Roxb.) Craib
*Kalopanax pictus* (Thunb.) Nakai	*Acer pictum* Thunb.
*Ledebouriella divaricate* (Turcz.) Hiroë	*Saposhnikovia divaricate* (Turcz.) Schischk.
*Lespedeza cuneata* (Dum. Cours.) G. Don	*Lespedeza juncea* (L. f.) Pers.
*Lindera glauca* (Siebold & Zucc.) Blume	*Lindera communis* Hemsl.
*Litsea sebifera* Pers.	*Litsea glutinosa* (Lour.) C.B. Rob.
*Loranthus parasiticus* (L.) Merr.	*Scurrula parasitica* L.
*Madhuca indica* J.F. Gmel.	*Madhuca longifolia* (J. Koenig ex L.) J.F. Macbr.
*Magnolia fargesii* (Finet & Gagnep.) W.C. Cheng	*Magnolia biondii* Pamp.
*Margyricarpus setosus* Ruiz & Pav.	*Margyricarpus pinnatus* (Lam.) Kuntze
*Maytenus heterophylla* (Eckl. & Zeyh.) N. Robson	*Gymnosporia heterophylla* (Eckl. & Zeyh.) Loes.
*Maytenus senegalensis* (Lam.) Exell	*Gymnosporia senegalensis* (Lam.) Loes.
*Melandrium seoulense* (Nakai) Nakai	*Silene seoulensis* Nakai
*Mentha haplocalyx* Briq.	*Mentha canadensis* L.
*Mosla punctulata* (J.F. Gmel.) Nakai	*Mosla scabra* (Thunb.) C.Y. Wu & H.W. Li
*Mutisia viciifolia* fo. *intermedia* Cuatrec.	*Mutisia acuminata* Ruiz & Pav.
*Orthosiphon labiatus* N.E. Br.	*Ocimum labiatum* (N.E. Br.) A.J. Paton
*Persicaria senticosa* (Meisn.) H. Gross ex Nakai	*Polygonum senticosum* (Meisn.) Franch. & Sav.
*Peucedanum graveolens* (L.) Hiern	*Anethum graveolens* L.
*Phoradendron juniperinum* Engelm. ex A. Gray	*Phoradendron ligatum* Trel.
*Polanisia icosandra* (L.) Wight & Arn.	*Cleome viscosa* L.
*Polygonum cuspidatum* Sieb. et Zucc.	*Reynoutria japonica* Houtt.
*Polygonum multiflorum* (Meisn.) H. Gross ex Nakai	*Reynoutria multiflora* (Thunb.) Moldenke
*Pongamia glabra* Vent.	*Pongamia pinnata* (L.) Pierre
*Pulsatilla chinensis* (Bunge) Regel	*Anemone chinensis* Bunge
*Quercus pedunculata* Hoffm.	*Quercus robur* L.
*Rhodiola rosea* L.	*Sedum rosea* (L.) Scop.
*Rhus acuminata* DC.	*Toxicodendron acuminatum* (DC.) C.Y. Wu & T.L. Ming
*Rhus javanica* L.	*Brucea javanica* (L.) Merr.
*Rhodiola rosea* L.	*Sedum rosea* (L.) Scop.
*Rumex bequaertii* De Wild.	*Rumex nepalensis* Spreng.
*Rumex cuneifolius* Campd.	*Rumex frutescens* Thouars
*Sapium japonicum* (Siebold & Zucc.) Pax & K. Hoffm.	*Neoshirakia japonica* (Siebold & Zucc.) Esser
*Satureja boliviana* (Benth.) Briq.	*Clinopodium bolivianum* (Benth.) Kuntze
*Scrophularia koraiensis* Nakai	*Scrophularia kakudensis* Franch.
*Senecio culcitioides* Sch. Bip.	*Senecio comosus* Sch. Bip.
*Scutellaria baicalensis* Georgi	*Scutellaria macrantha* Fisch. ex Rchb.
*Sophora angustifolia* Siebold & Zucc.	*Sophora flavescens* Aiton
*Sophora japonica* L.	*Styphnolobium japonicum* (L.) Schott
*Sophora subprostrata* Chun & T.C. Chen	*Sophora tonkinensis* Gagnep.
*Syringa dilatata* Nakai	*Syringa oblata* var. *dilatata* (Nakai) Rehder
*Teclea simplicifolia* (Engl.) I. Verd.	*Vepris simplicifolia* (Engl.) Mziray
*Tinospora cordifolia* (Willd.) Miers ex Hook. f. & Thomson	*Tinospora sinensis* (Lour.) Merr.
*Trigonostemon lii* Y.T. Chang	*Trigonostemon bonianus* Gagnep.
*Tripterygium hypoglaucum* (H. Lév.) Hutch.	*Tripterygium wilfordii* Hook. f.
*Tulipa edulis* (Miq.) Baker	*Amana edulis* (Miq.) Honda
*Veronica linariifolia* Pall. ex Link	*Pseudolysimachion linariifolium* (Pall. ex Link) Holub
*Wedelia chinensis* (Osbeck) Merr.	*Sphagneticola calendulacea* (L.) Pruski
*Werneria ciliolate* A. Gray	*Xenophyllum ciliolatum* (A. Gray) V.A. Funk
*Werneria dactylophylla* Sch. Bip.	*Xenophyllum dactylophyllum* (Sch. Bip.) V.A. Funk
*Woodfordia floribunda* Salisb.	*Woodfordia fruticosa* (L.) Kurz
